# Supplementary material for: Structural and Dynamic Disorder, Not Ionic Trapping, Controls Charge Transport in Highly Doped Conducting Polymers
Source: J Am Chem Soc. 2022 Feb 14;144(7):3005–19. doi: 10.1021/jacs.1c10651 (PMC8874922; doi:10.1021/jacs.1c10651)
Supplement: Supplementary file 1 — ja1c10651_si_001.pdf [file ja1c10651_si_001.pdf]

Supporting Information for

Structural and dynamic disorder, not ionic trapping, controls charge transport in highly doped conducting polymers

Ian E. Jacobs, Gabriele D'Avino, Vincent Lemaur, Yue Lin, Yuxuan Huang, Chen Chen, Thomas Harrelson, William Wood, Leszek J. Spalek, Tarig Mustafa, Christopher A. O'Keefe, Xinglong Ren, Dimitrios Simatos, Dion Tjhe, Martin Statz, Joseph W. Strzalka, Jin-Kyun Lee, Iain McCulloch, Simone Fratini, David Beljonne, Henning Sirringhaus

January 2, 2022

This PDF file includes:
Figures S1-S39, Tables S1-S6, and supporting discussion

1 Exchange efficiency characterization

1.1 Ion dependent exchange efficiency

To understand how the identity of the polymer and ion affect the ion exchange efficiency, we measured UV-vis spectra of each polymer after a relatively long ion exchange doping time (5 min, 100/1 mM electrolyte/$FeCl_3$, AN). This longer doping time was chosen as a compromise between ensuring sufficient time for ion exchange for large ions, and avoiding irreversible oxidation reactions which we previously identified as a concern.[1] Figure S1 shows these spectra. Using the fitting routine described in Section 1.2 we then extracted the residual $FeCl_4^-$ concentration; these results are shown in Figure S2. In IDTBT, the conductivity is strongly dependent on doping level (see Section 3); the spectra and ion exchange efficiencies shown here correspond to the doping time which gave maximum conductivity. Doping time dependent exchange efficiencies for IDTBT are shown in Figure S4.

In both PBTTT and P3HT there is growing evidence that dopant ions are typically located in the sidechain region, consistent with our DFT simulations.[2–4] The sidechains of PBTTT are generally understood to be interdigitated, while those of P3HT are non-interdigitated and disordered.[5] This creates in PBTTT a well defined void space which can be populated by a guest molecule[6] or dopant ion. In our results, this difference between the structure of the side chain regions of PBTTT and P3HT manifests as a marked difference in the exchange efficiency for large ions.

In PBTTT, when the ion exceeds the size of the void (e.g. BArF) the exchange efficiency drops dramatically. As discussed in our previous work,[1] the free energy of the $FeCl_4^-$ to TFSI exchange reaction in PBTTT, $\Delta G_e x$, is 10-15% of the terrace phase crystallization enthalpy. To achieve the exchange efficiency of less than 5% observed for BArF, $\Delta G_e x$ would need to be equal to or greater than the crystallization enthalpy, suggesting that BArF is unable to incorporate into PBTTT crystallites.

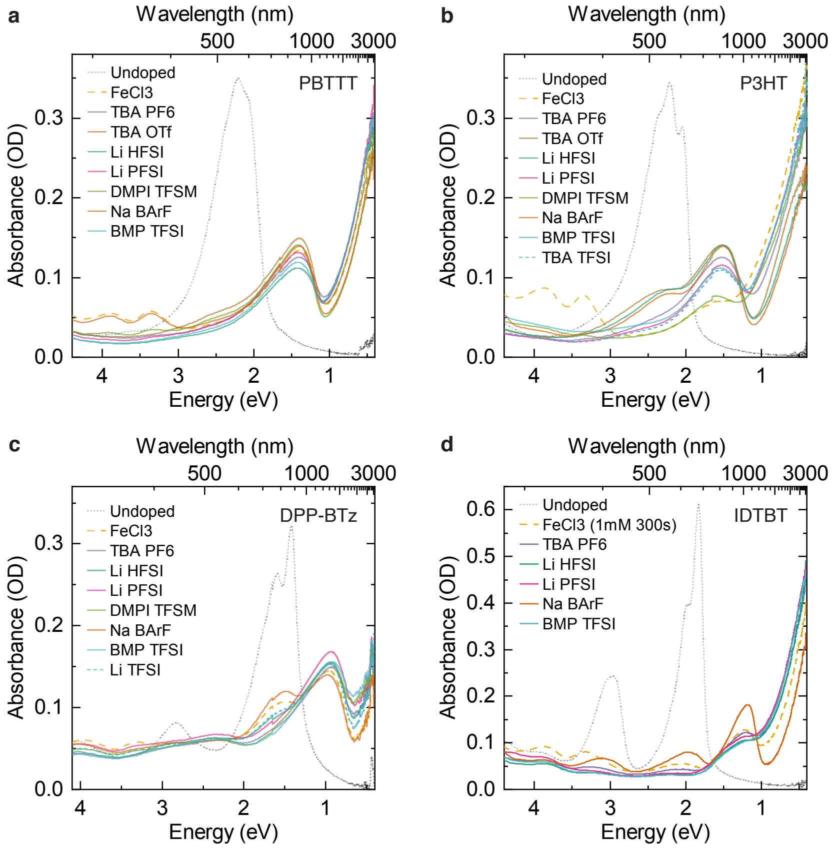

Figure S1: **UV-vis-NIR spectra of PBTTT, P3HT, DPP-BTz, and IDTBT doped by ion exchange**. All samples except for IDTBT were doped for 5 minutes (100/1 mM electrolyte/$FeCl_3$, AN). Spectra for IDTBT correspond to the maximum conductivity samples, full time-dependent spectra and further discussion are given in Section 3.

Comparing the results for TFSI, HFSI, and PFSI, we can also clearly see that the shape of the ion affects the exchange efficiency. In our optimized 1:1 mole ratio DFT structure (Figure 3c, main paper), the TFSI ion is forced into an 'L' shape because the length of the extended TFSI ion is greater than the size of the unit cell. The HFSI ion is effectively a TFSI ion with the two CF_3 groups bridged by a CF_2 group to form a ring. We expect that shorter length of HFSI should reduce the energetic barrier required to reach high doping levels, and likely explains why we observe a somewhat higher doping level for HFSI in the UV-vis spectra of doped PBTTT (Figure S1); Figure 1c, main paper). On the other hand PFSI, which is structurally similar to but longer than TFSI, would be expected to distort into a non-linear geometry at lower doping levels. As a result, we observe an even longer lamellar stacking distance, lower exchange efficiency, and slightly lower doping levels for PFSI (Figure S3).

In P3HT, the side chain structure is more liquid-like and voids are poorly defined.[5]. There is little energetic cost associated with disrupting this already disordered structure, therefore the exchange reaction free energy ΔG^0_{ex} may be significantly greater than the polymer enthalpy of crystallization, and therefore capable of severely disrupting the polymer microstructure. In this regime, exchange efficiencies are high even for large ions such as BArF, but crystallinity may drop significantly depending on the ability of the polymer and ion to co-crystallize in an ordered structure. In P3HT this manifests as a complex interplay between doping level, paracrystallinity, and lamellar stacking distance for different ions.

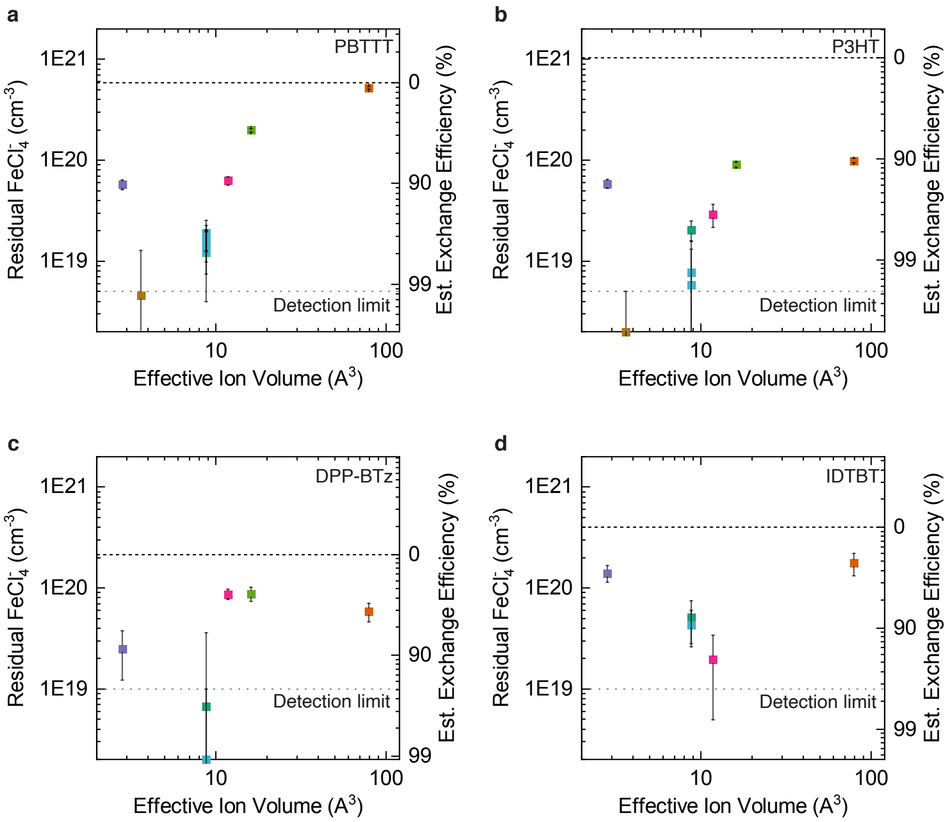

Figure S 2: **Residual $FeCl_4^-$ in PBTTT, P3HT, DPP-BTz, and IDTBT doped by ion exchange**. All samples except for IDTBT were doped for 5 minutes (100/1 mM electrolyte/$FeCl_3$, AN). Data for IDTBT correspond to the maximum conductivity samples, full time-dependent data and further discussion are given in Section 3.

The strong effect of doping on microstructure is clearest in P3HT:TFSM, which displays a very high doping level, short lamellar stacking distance, and nearly amorphous microstructure (Section 4.2). The very short lamellar stacking distance—shorter even than the undoped polymer—along with the almost complete disappearance of the $\pi - \pi$ stacking reflection in GIWAXS is consistent with incorporation of TFSM into the polymer π-stacking region, rather than the sidechain. Similar behavior has previously been observed in P3HT:F4TCNQ.[7] Both TFSI and PFSI also show shorter lamellar stacking distances than $FeCl_4^-$ and paracrystallinities 3-4% greater than undoped P3HT. In the context of P3HT:TFSM, it therefore appears likely that both TFSI and PFSI are to some degree intercalated into the P3HT π-stacking region.

We also observe some variability in doping level between ions in P3HT—BArF and HFSI both show some residual $\pi - \pi^*$ absorbance in UV-vis spectra (Figure S1), while TFSM and $FeCl_4$ both show almost complete bleaching of the P2 band. Although it is plausible that differences in dilute water content could lead to variation in the Fe^{3+} reduction potential,[1] we would expect this mechanism results in a systematic effect across all polymers. A more likely possibility is a structural effect, where the overall doping reaction ΔG is in part controlled by the molecular packing of the polymer and dopant ion. This theory is consistent with the lower doping levels observed for HFSI and BArF, which may derive from an inability of these ions to incorporate into the π-stacking region, consistent with their larger lamellar stacking distances. Further work will clearly be required to fully understand the structure-property relationships of ion exchange doped P3HT. We stress that in the doping process described here, we do not observe any evidence that the cation

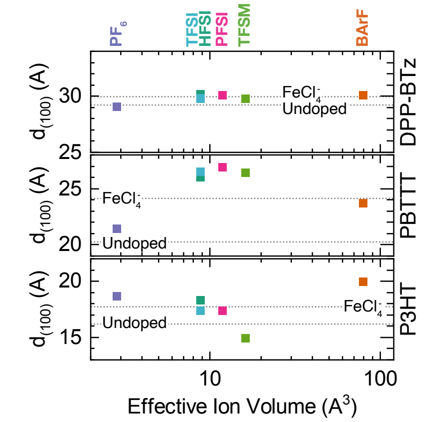

Figure S3: **Lamellar stacking distances in PBTTT, P3HT, DPP-BTz, for various dopant ions**. All samples were doped for 5 minutes (100/1 mM electrolyte/$FeCl_3$, AN). Y axis range is identical for all plots to allow for visual comparison of the magnitude of the changes in stacking distance. Effective ionic volume is defined as $\frac{4\pi}{3}\lambda_x\lambda_y\lambda_z$, where λ_i are the principal moments of the ionic gyration tensor. Full GIWAXS results are given in Section 4.

affects the exchange efficiency, in line with our previous findings[1] but in contrast with the observations of Yamashita et al.[8] We attribute this difference to the high dielectric constant of the doping solvent used here (acetonitrile, $\epsilon_r \approx 36.7$)[9] which is expected to eliminate the ion-association effects that were proposed to limit the exchange efficiency in Yamashita et al. A full discussion of these effects are given in Ref 1.

In DPP-BTz, we observe relatively low maximum doping levels (3.05×10^{20} cm^{-3} with HFSI by XPS; see Figure S14). With PF_6, TFSI, and HFSI exchange efficiencies are quite high, however for larger ions the efficiency drops to 60-70%. Interestingly, here the lamellar stacking distance varies little between the undoped polymer and each ion. This is perhaps indicative of preferential segregation of ions to amorphous regions. However our lack of a clear picture of molecular packing in DPP-BTz limits our ability to interpret these results.

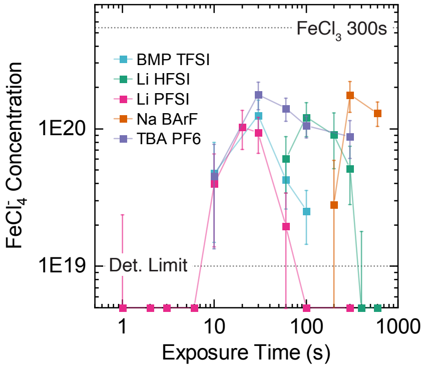

Figure S4: **Doping time dependent ion exchange efficiency in IDTBT**. All samples were doped with 100/1 mM electrolyte/$FeCl_3$, AN. Time dependent UV-vis spectra and conductivity are shown in Section 3.

As noted above and discussed in further detail in Section 3, the conductivity of doped IDTBT is strongly dependent on doping time. For long doping times, residual $FeCl_4^-$ concentrations are quite low (Figure S4). Given the largely amorphous microstructure of IDTBT (Section 4.4), these high exchange efficiencies corroborate our conclusion that ΔG^0_{ex} is largely controlled by the energy required to distort the polymer crystal to incorporate the dopant ion. While high exchange efficiencies are possible in IDTBT even with larger ions such as PFSI, the time required for efficient exchange in most cases appears to be longer than the doping time required to reach the optimum carrier density. As a result, the exchange efficiency at maximum conductivity (Figure S2d) is lower than it might be if the doping process was divided into two separate steps—doping, followed by ion exchange, as previously suggested.[10,11] Our measurement of the carrier density in maximum conductivity IDTBT films is taken from a 1 mM $FeCl_3$ doped film (shown in Figure S1d) which shows slightly lower doping level than the the optimally doped films (with the exception of BArF). Therefore, the exchange efficiencies estimated in Figure S2)d are relatively conservative estimates.

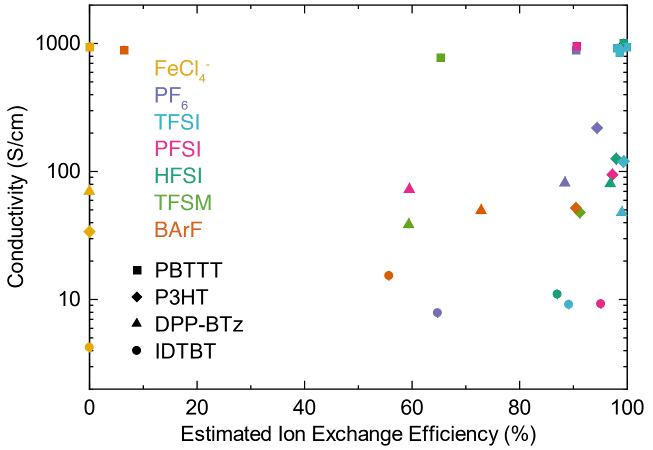

Figure S5: **Effect of residual $FeCl_4^-$ on electrical conductivity**. Plot of estimated ion exchange efficiency vs. electrical conductivity. Ion exchange efficiency is estimated from the observed $FeCl_4^-$ concentration in each ion exchange sample and the $FeCl_4^-$ concentration measured for $FeCl_3$ doped samples without ion exchange. True exchange efficiencies are likely to be somewhat higher, as the carrier density in ion exchange samples is likely higher due to the additional entropic driving force for exchange.

Although in most samples the exchange efficiency exceeds 90%, in a few polymer:ion combinations the observed exchange efficiency is low as ∼60%. To verify that this non-ideal exchange does not affect our results, we plot the electrical conductivity vs. the estimated ion exchange efficiency in Figure 5. We do not observe any clear correlation between exchange efficiency and electrical conductivity in the dataset as a whole, or within each polymer individually. This observation suggests that the presence of $FeCl_4^-$ impurities do not significantly affect charge transport within the material, consistent with our general findings that ionic size (and consequently variation in ionic size due to $FeCl_4^-$ impurities) has little effect on electrical conductivity at these high doping levels.

1.2 Fitting residual $FeCl_4^-$ concentration spectroscopically

The concentration of $FeCl_4^-$ remaining in the film after ion exchange can be fit spectroscopically due to the sharp UV absorption features of the $FeCl_4^-$ anion in the UV. We follow the same fitting procedure used in our previous study,[1] using the following expression for the absorbance of the UV region:

Table S 1: $FeCl_4^-$ UV spectral fit details

Figure	Polymer	Doping time	Fit range (eV)	Baseline function	δ range (eV)
Figure S6	PBTTT	5 min.	$2.8 < E < 4.3$	Poly. (3rd order)	$-0.07 < \delta < -0.06$
Figure S7	P3HT	5 min.	$3.0 < E < 4.2$	Poly. (3rd order)	$-0.10 < \delta < -0.09$
Figures S8	DPP-BTz	5 min.	$2.4 < E < 4.13$	Poly. (2rd order) + Undoped spectrum + Gaussian (4.07 eV)	$-0.10 < \delta < -0.09$
Figures S9-13	IDTBT	Variable (t-dep)	$2.5 < E < 4.4$	Poly. (3rd order) + Undoped spectrum + Gaussian (3.85 eV)	$-0.085 < \delta < -0.07$

$$A(E) = \epsilon(E - \delta) l_f c + B(E) \tag{1}$$

where l_f is the film thickness and δ account for the solvatochromic shift of the $FeCl_4^-$ due to the difference in the dielectric constant of the medium (i.e. polymer or solvent). $B(E)$ is a baseline function accounting for the residual absorbance of the polymer in the UV:

$$B(E) = \sum_{i=0}^{n} c_i E^i + c_u A_u(E) \tag{2}$$

where c_i are fit coefficients and $A_u(E)$ is the undoped polymer absorption. The inclusion of the undoped polymer absorption in the baseline function is required in the D-A copolymers IDTBT and DPP-BTz because the strong UV absorption band in the undoped polymers appeared in the fits even for nearly fully doped polymers. In IDTBT and DPP-BTz we also found that it was necessary to include a weak gaussian absorption deeper in the UV region (3.8 - 4.1 eV) to obtain good fits. This absorption peak is quite low in intensity, on the order of 0.02 OD, and could result from many things; for instance a weak $\sigma - \sigma*$ transition or a higher energy polaronic excited state. Table S1 provides full details of the parameters used in fitting each set of spectra; carrier densities calculated from fits to films doped with $FeCl_3$ without ion exchange are presented in Table S2 along with values obtained from XPS and NMR.

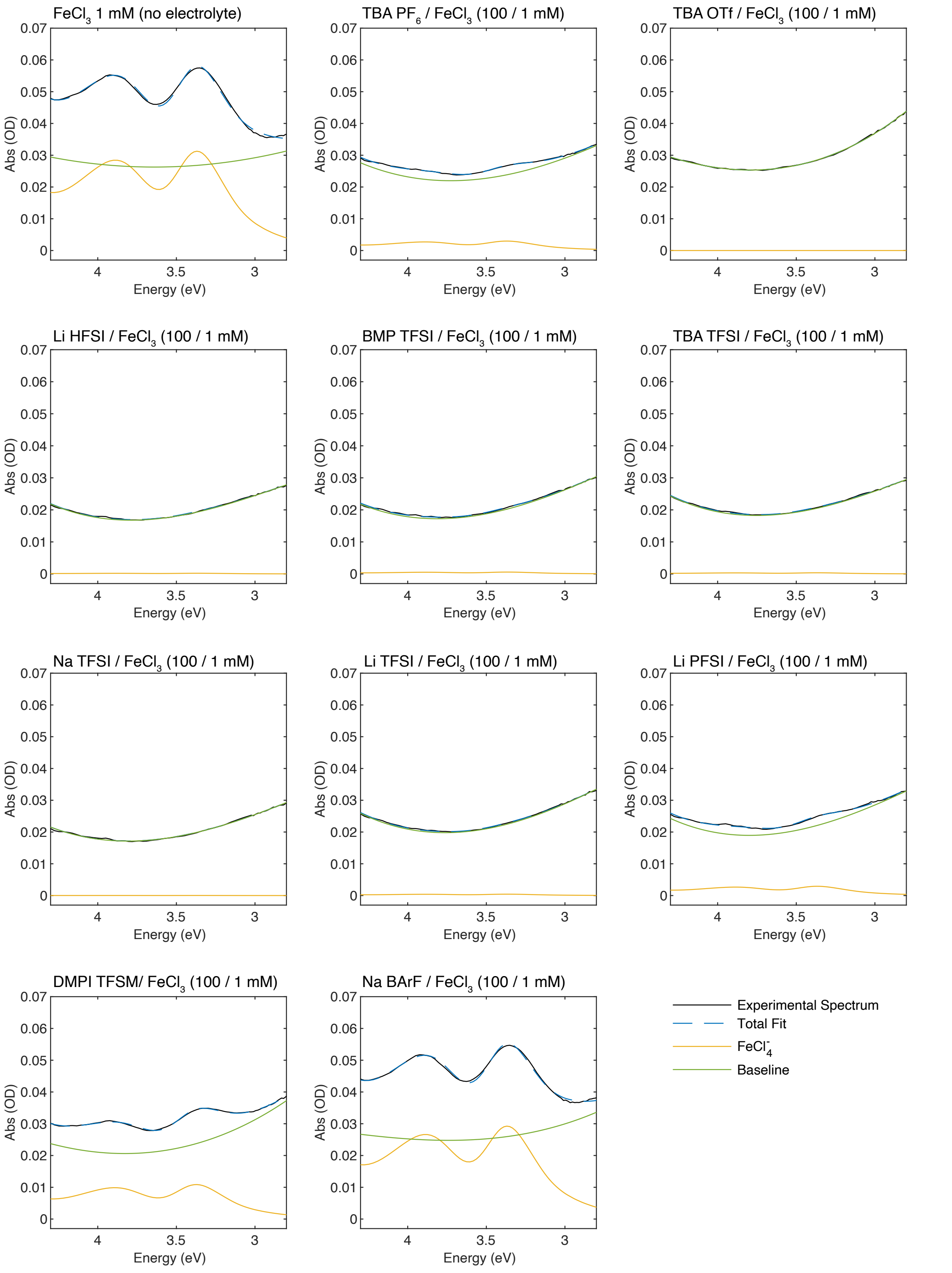

Figure S6: **PBTTT ion dependent UV fits, 5 min. doping time (max. doping level)**. Fits to UV-vis spectra of ion exchange doped PBTTT films. Doping solutions contained 1 mM $FeCl_3$ and 100 mM electrolyte (see label above each plot). For fit details see Table S1.

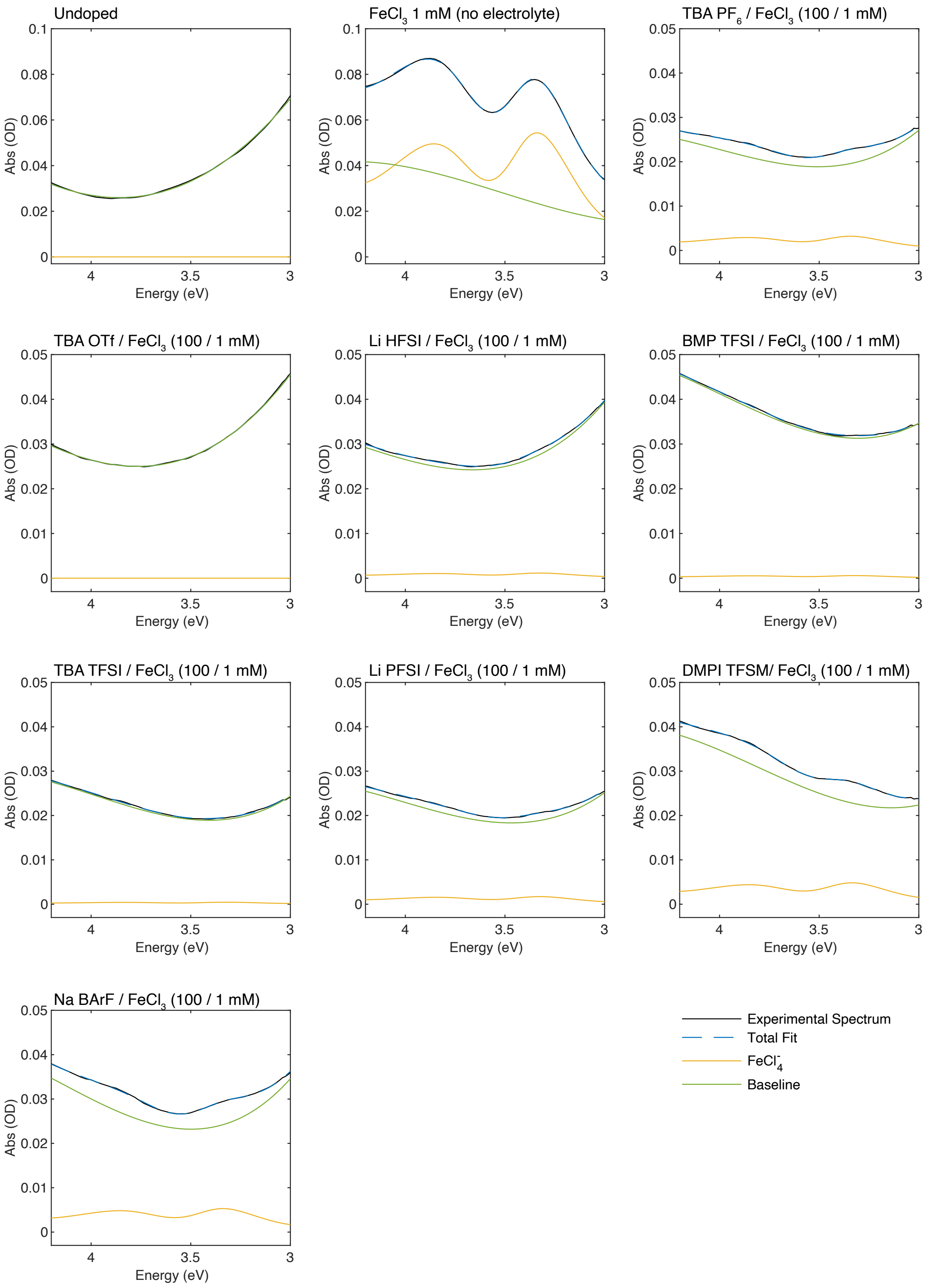

Figure S7: **P3HT ion dependent UV fits, 5 min. doping time (max. doping level)**. Fits to UV-vis spectra of ion exchange doped P3HT films. Doping solutions contained 1 mM $FeCl_3$ and 100 mM electrolyte (see label above each plot). For fit details see Table S1.

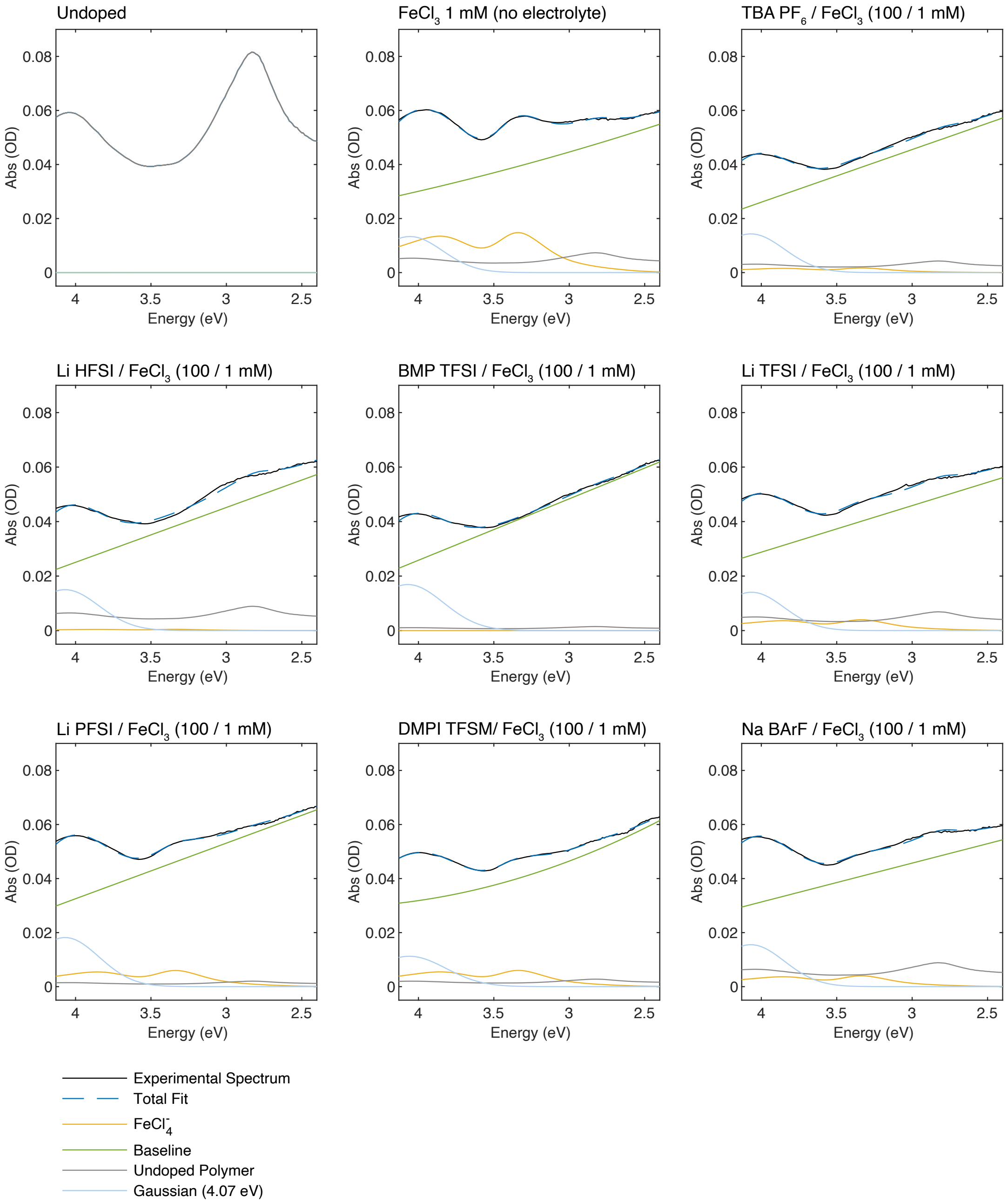

Figure S8: **DPP-BTz ion dependent UV fits, 5 min. doping time (max. doping level)**. Fits to UV-vis spectra of ion exchange doped DPP-BTz films. Doping solutions contained 1 mM $FeCl_3$ and 100 mM electrolyte (see label above each plot). For fit details see Table S1.

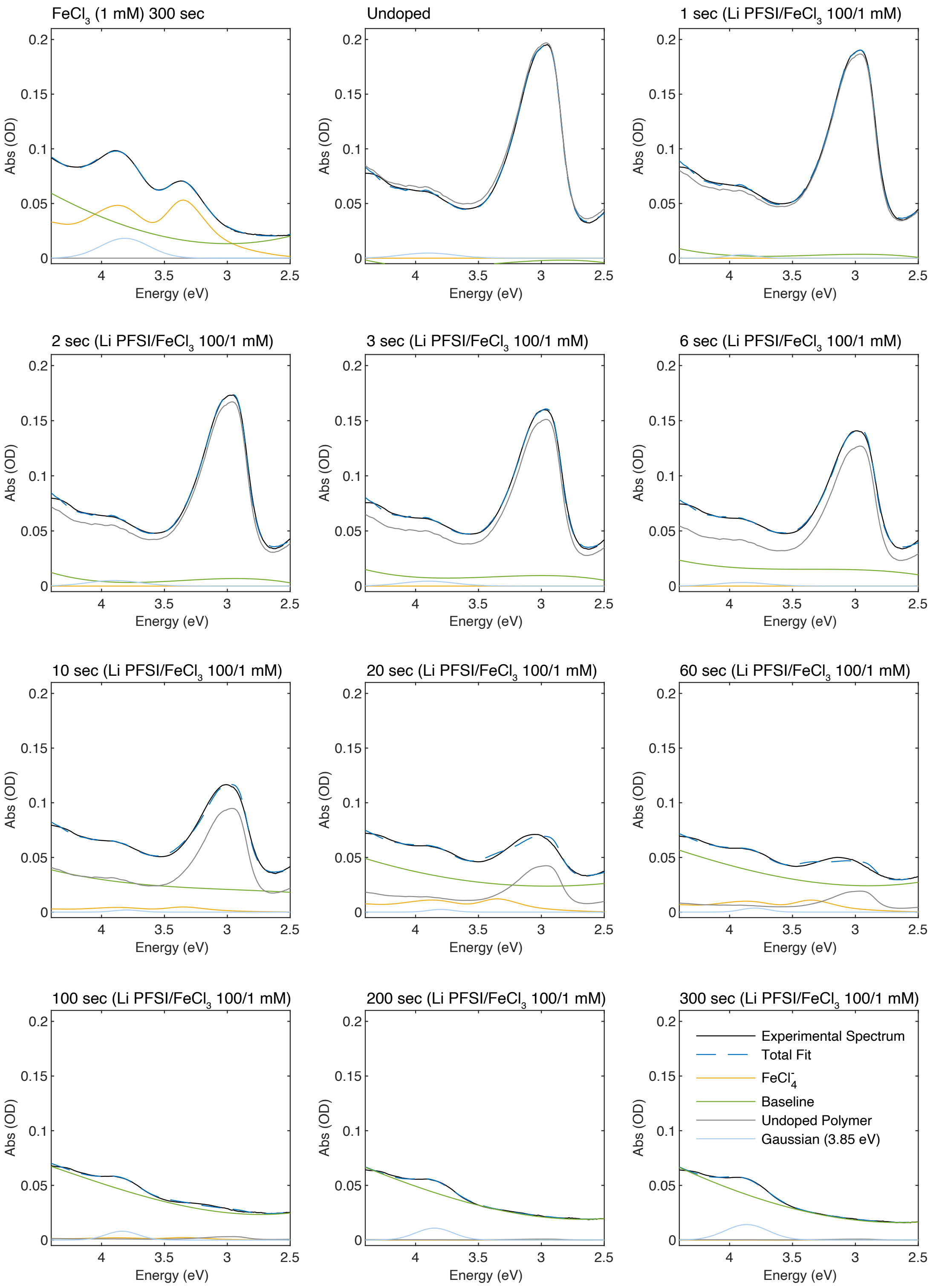

Figure S9: **IDTBT : Li PFSI UV-vis fits, variable doping time**. Fits to UV-vis spectra of ion exchange doped IDTBT films. Doping solutions contained 1 mM $FeCl_3$ and 100 mM electrolyte; see label above each plot for doping time. For fit details see Table S1.

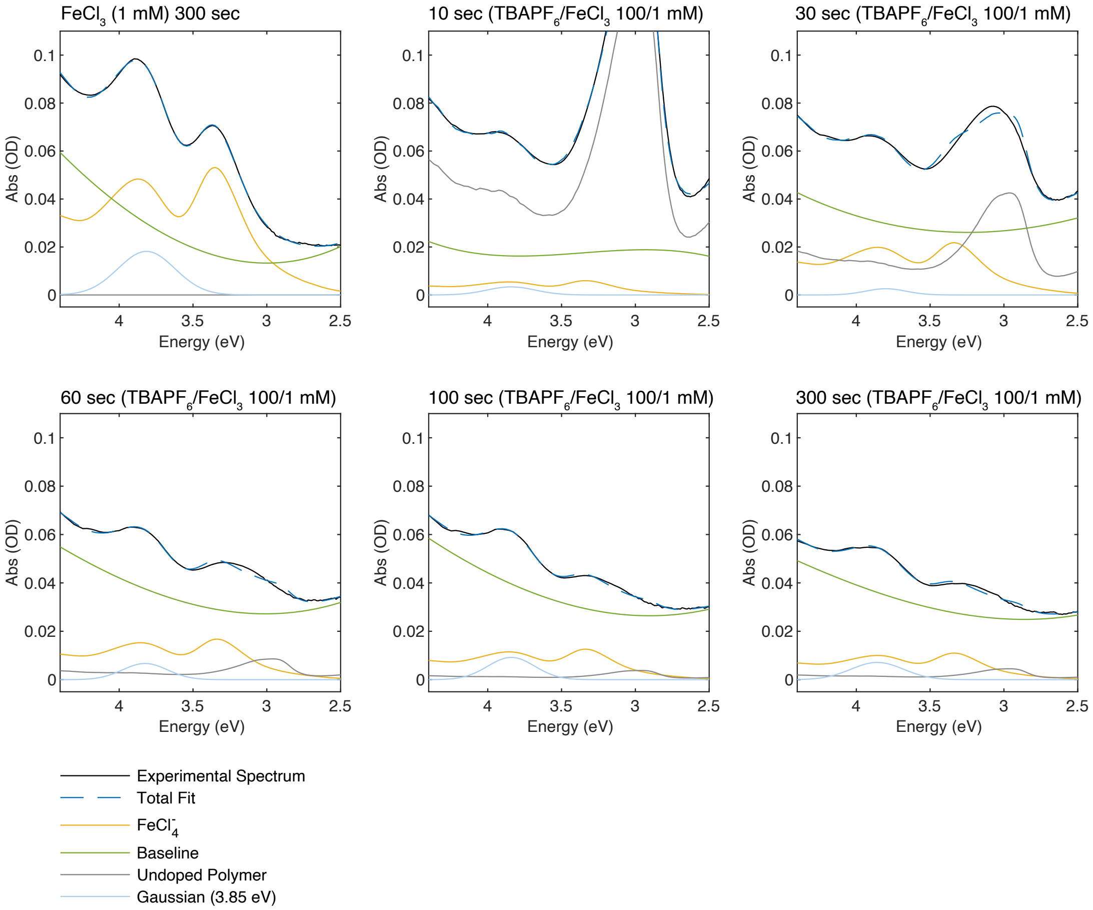

Figure S10: **IDTBT : TBA PF_6 UV-vis fits, variable doping time**. Fits to UV-vis spectra of ion exchange doped IDTBT films. Doping solutions contained 1 mM $FeCl_3$ and 100 mM electrolyte; see label above each plot for doping time. For fit details see Table S1.

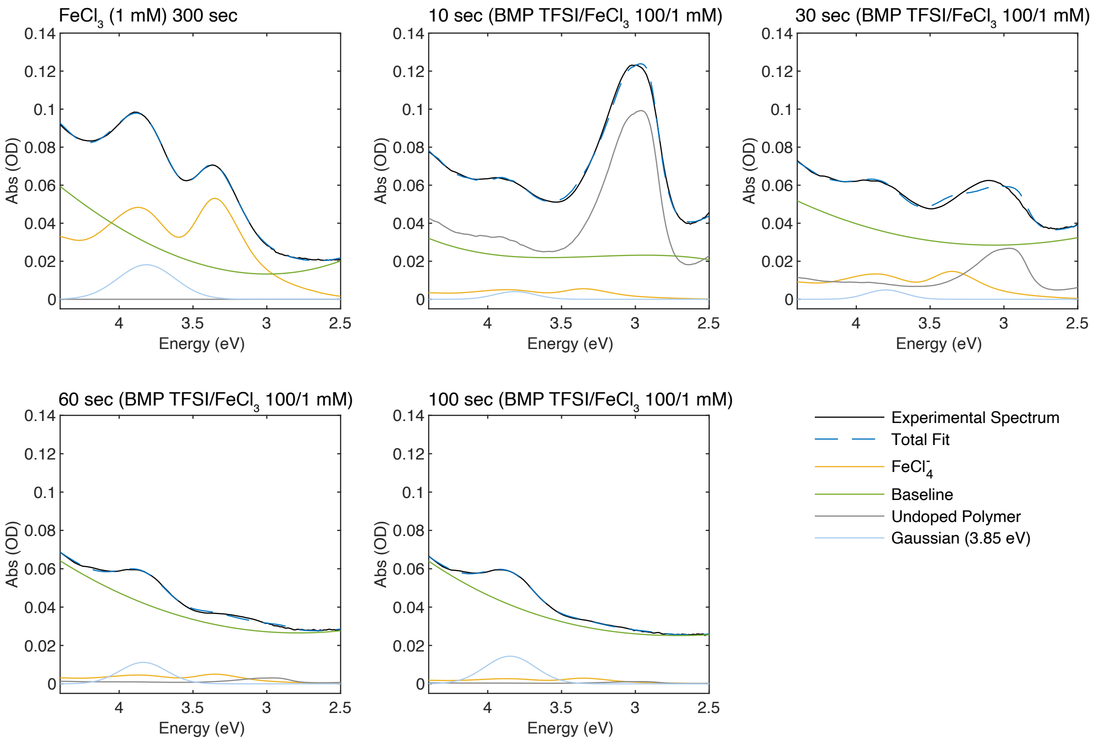

Figure S11: **IDTBT : BMP TFSI UV-vis fits, variable doping time**. Fits to UV-vis spectra of ion exchange doped IDTBT films. Doping solutions contained 1 mM $FeCl_3$ and 100 mM electrolyte; see label above each plot for doping time. For fit details see Table S1.

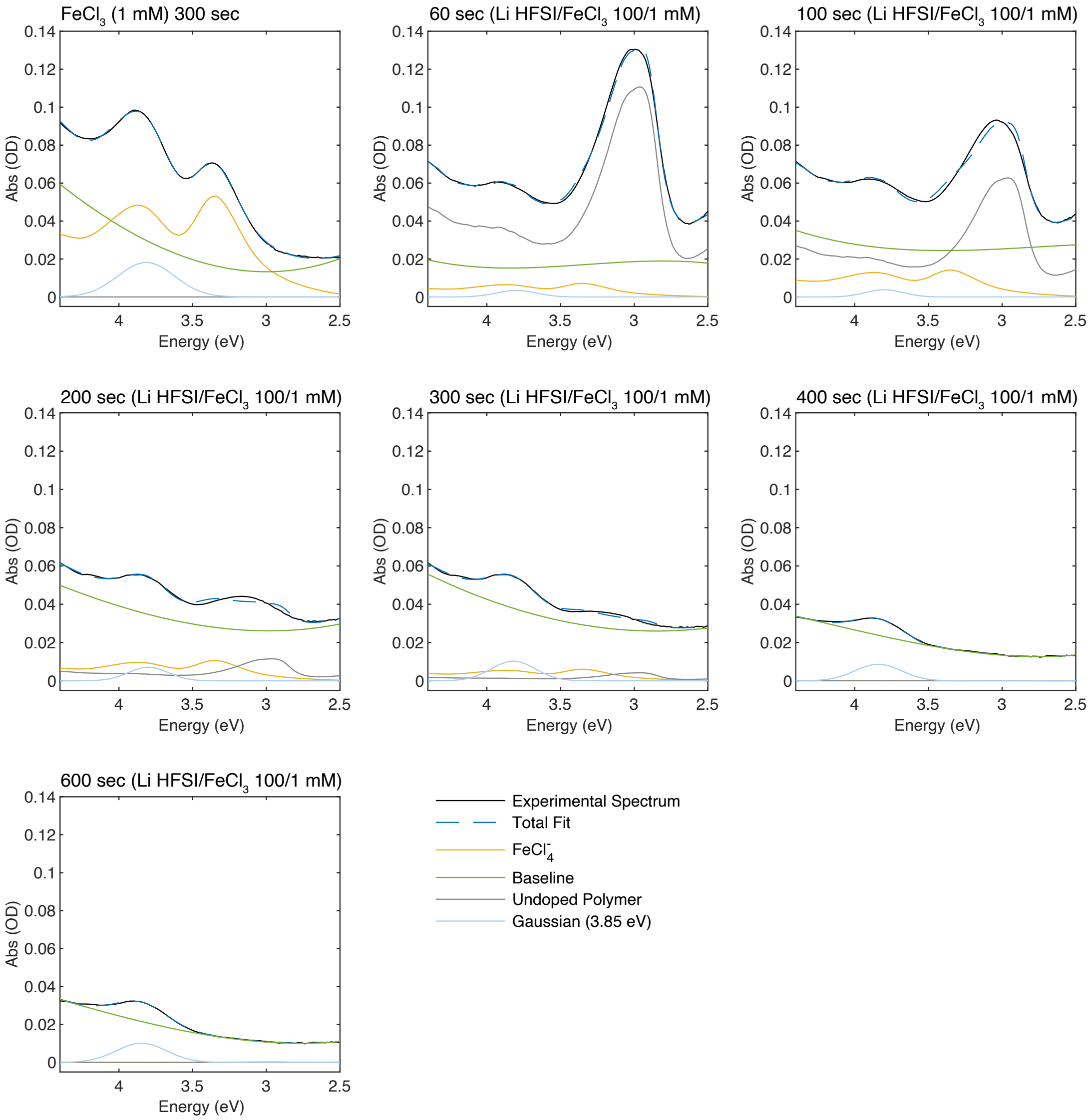

Figure S12: **IDTBT : Li HFSI UV-vis fits, variable doping time**. Fits to UV-vis spectra of ion exchange doped IDTBT films. Doping solutions contained 1 mM $FeCl_3$ and 100 mM electrolyte; see label above each plot for doping time. For fit details see Table S1.

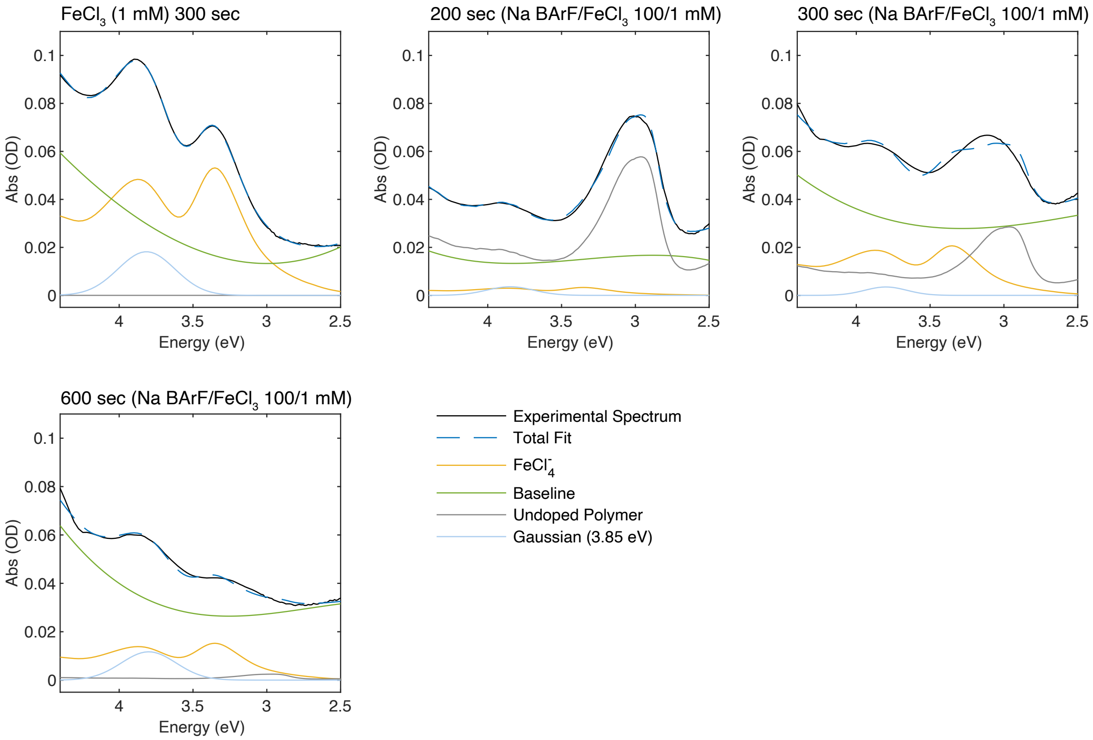

Figure S13: **IDTBT : Na BArF UV-vis fits, variable doping time**. Fits to UV-vis spectra of ion exchange doped IDTBT films. Doping solutions contained 1 mM $FeCl_3$ and 100 mM electrolyte; see label above each plot for doping time. For fit details see Table S1.

2 Carrier density measurement

2.1 X-ray photoemission spectroscopy

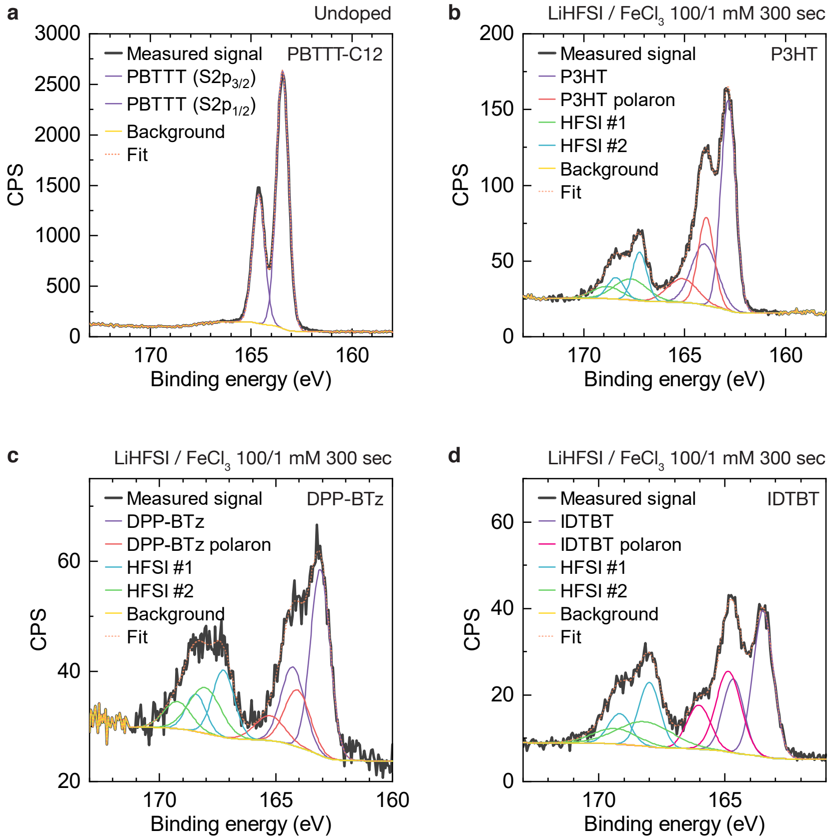

Figure S14: **Carrier density measurement by XPS**. Sulfur 2p XPS spectra of polymer films: a) PBTTT-C12 undoped. b) P3HT:HFSI doped with 100/1 mM Li HFSI / $FeCl_3$, 300s. c) DPP-BTz:HFSI doped with 100/1 mM Li HFSI / $FeCl_3$, 300s. d) IDTBT:HFSI doped with 100/1 mM Li HFSI / $FeCl_3$, 300s.

Figure S14 shows the XPS sulfur 2p spectra of undoped PBTTT-C12, P3HT:HFSI, DPP-BTz:HFSI, and IDTBT:HFSI, along with fits to the experimental spectra. HFSI was chosen for these measurements over TFSI as it gave among the highest conductivity of all ions studied in each polymer, achieved very high exchange efficiencies in all polymers (see Section 1.2), and contained sulfur atoms, allowing us to fit the concentration from a single measurement of the sulfur XPS spectrum.

We begin our analysis by observing that the sulfur XPS spectrum of undoped PBTTT (Figure 14a) can be fit with a single S2p doublet, consisting of a $S2p_{3/2}$ and $S2p_{1/2}$ signal, with the $S2p_{1/2}$ shifted deeper in energy by the spin-orbit splitting (1.18 eV) and having half the area. The ability to fit the undoped spectrum with a single S2p doublet indicates that the thiophene and thienothiophene sulfur atoms in PBTTT are equivalent, at least to within the resolution of our instrument.[1] The second polymer-derived S2p doublet required to achieve satisfactory fits in doped PBTTT therefore cannot derive from chemical inequivalency

[1]Note that this measurement is of a PBTTT-C12 film, as opposed to PBTTT-C14 used elsewhere in this work. PBTTT-C12 was used here as the batch of PBTTT-C14 was finished prior to this measurement. However, we do not expect that the polymer side chain length should affect the equivalency of the backbone sulfur atoms.

of the sulfur atoms within PBTTT, but rather must originate from an increase in binding energy for sulfur atoms in the presence of polarons. Winkler et al. reached a similar conclusion in their detailed photoemission measurements of C60 hole polarons.[12]

The fitting routine for all doped polymers studied here is similar to those presented for PBTTT:TFSI. In all cases, the signals of the polymer and the ion are chemically shifted by the large differences in electron density of the p-doped polymer vs. anion, allowing for relatively straightforward separation of the signal from each species. In all cases the anion signal, appearing at higher binding energy, required two inequivalent doublet peaks to fit, as in the PBTTT:TFSI fits, suggesting an inequivalent environment for the two S atoms of the HFSI. This observation suggests that the ion may pack with one the SO_2 groups located closer to the polymer backbone than the other, likely as a way of reducing the electrostatic interaction between the two molecules.

In fitting our XPS data, we assume that the ratio of the two polymer signals XPS (neutral and polaron) correspond to the degree of oxidation of the backbone, δ, with full oxidation ($\delta = 1$; complete bleaching of the neutral signal) corresponding to a $2e^-$ oxidation of each monomer, i.e. a bipolaron on each site. At doping levels of around 1 ion per monomer ($\delta = 0.5$, as observed in most polymers studied here, we therefore observe two polymer S2p signals with approximately equal areas. The fit function is given by

$$S(E) = (1-\delta)n_{poly}I_0V(E;\sigma,\gamma) + \delta n_{poly}I_0V(E;\sigma,\gamma) + 2\delta n_{ion,1}I_0V(E;\sigma,\gamma) + 2\delta n_{ion,2}I_0V(E;\sigma,\gamma) \quad (3)$$

where n_{poly} is the number of equivalent sulfur atoms per monomer, $n_{ion,i}$ is the number of equivalent sulfur atoms contributing to the ith ion signal, $V(E;\sigma,\gamma)$ is a Voigt function with width σ and lineshape parameter γ, and I_0 is an arbitrary factor that scales $S(E)$ but does not affect peak ratios. The factor of 2 in the ion signal (last two terms) enforces the assumption given above that full oxidation corresponds to $2e^-$ per monomer. The Voigt function is normalized, therefore the peak areas depend only on the number of sulfur atoms contributing to each peak and the doping level. The molar doping level in ions per monomer is easily obtained from the fits as 2δ; these values are reported in Table 1 of the main paper.

In PBTTT, DPP-BTz, and IDTBT we obtain good fits with n_{poly} equal to the number of sulfur atoms per monomer, while in P3HT satisfactory fitting requires $n_{poly} = 4$. This suggests that polarons are delocalized over about 4 monomers in P3HT, consistent with recent IR spectroscopy studies that have similarly shown polaron delocalization over about 4 monomers in P3HT.[13] Note that while the choice of n_{poly} affects the ratio of the neutral and polaron bands within the polymer signal, it has relatively little effect on δ (i.e. the doping level) because the ion and polymer signals are quite well resolved. In this sense, while the model provided above gives us some insight into materials properties, the validity of these assumptions do not significantly affect the measured doping level.

In the spectra shown in Figure S14b-d, the signal to noise was degraded significantly due to issues with the focus of the X-ray beam in our instrument; the PBTTT spectra were collected earlier before this problem appeared. This increase in noise did not strongly increase the size of the error bars, however it did necessitate a much longer measurement time than used in the PBTTT samples (170 scans vs. 30 scans). To verify that this longer beam exposure did not lead to beam damage, we also fit the low resolution S spectra obtained during survey scans collected prior to the high resolution measurements. In P3HT these measurements gave consistent values, while in DPP-BTz and IDTBT the survey scan gave values about 10% higher than obtained from the high-resolution spectra. This suggests that some dedoping may occur during long XPS measurements of these samples, and that the values presented in Table S2 may be slight underestimates of the true carrier density for DPP-BTz and IDTBT.

2.2 Quantitative NMR

Figure S15b shows ^{19}F spectra of TFSI extracted with a 10% triethylamine / CD_3CN solution from PBTTT:TFSI films doped for 100 or 300 seconds, as well as the spectrum of a the 100s doped film washed with pure CD_3CN. To verify that dedoping was complete, and thus that nearly all TFSI ions in the film had been extracted into the NMR solution, UV-vis spectra were collected after dedoping the films (Figure S15d) revealing nearly complete recovery of the polymer $\pi - \pi^*$ absorption band (2-2.5 eV) and almost no remaining polaronic

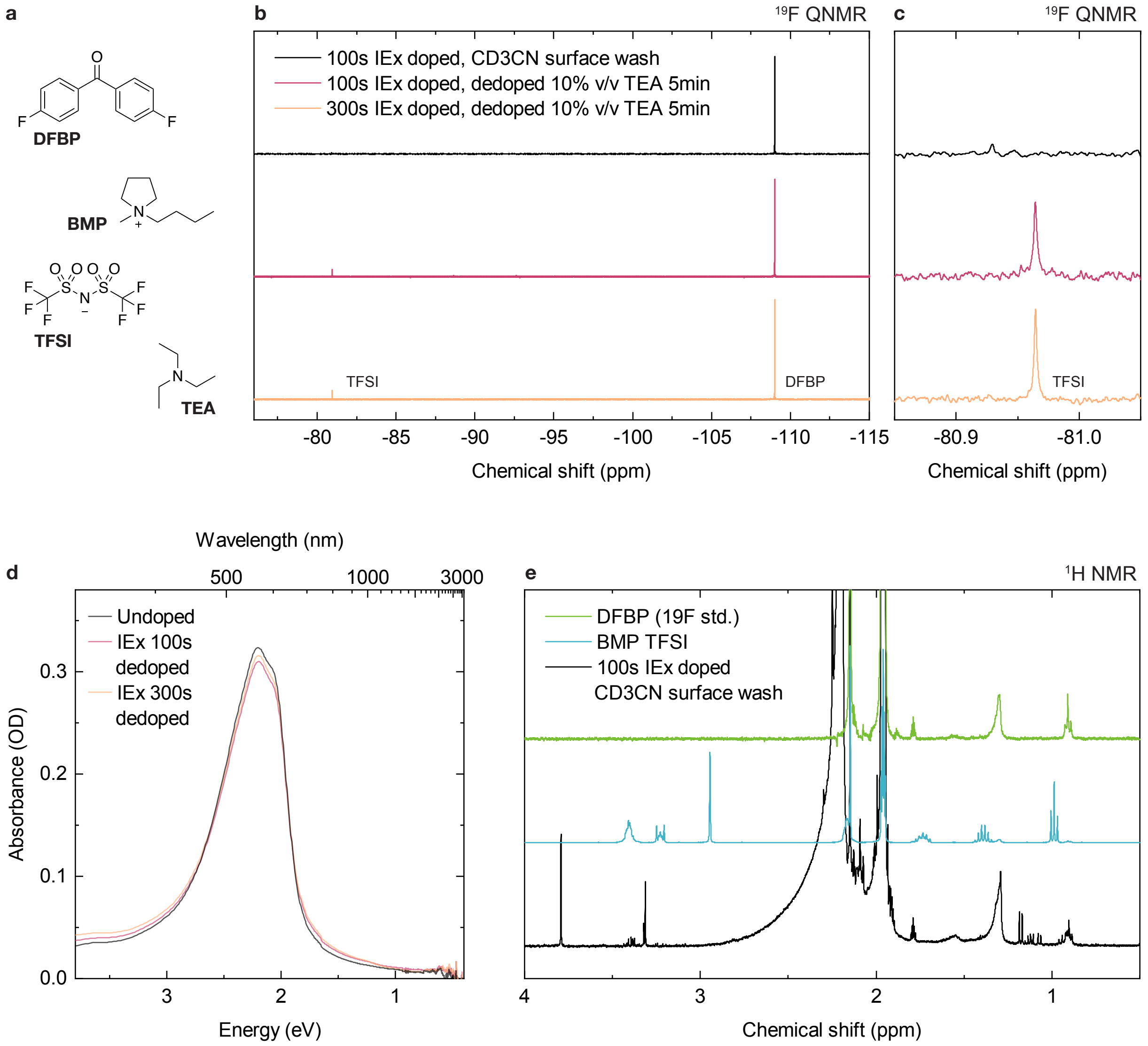

Figure S15: **Carrier density measurement in PBTTT:TFSI by quantitative NMR**. a) Molecular structures of 4,4-difluorobenzophenone (DFBP), triethylamine (TEA) and TFSI. b) ^{19}F QNMR spectrum of IEx doped PBTTT:TFSI films (100 or 300 sec exposure time, 100/1 mM BMP TFSI / $FeCl_3$ in AN) after either washing with CD_3CN, or dedoping wtih TEA (10% v/v in CD_3CN, 5 min). c) Detail of (b) showing TFSI peak. d) UV-vis-NIR spectra of films used in (b) after dedoping, compared with an undoped film prepared contemporaneously. e) ^{1}H NMR spectra of DFBP, BMP TFSI, and a surface washed IEx doped film, verifying the absence of BMP cations.

absorptions (1.5 eV and <0.5 eV), indicative of effectively complete dedoping.[14] The slight residual $\pi - \pi^*$ bleaching in the dedoped samples relative to the undoped film is likely a result of a small fraction of irreversibly oxidized polymer sites generated during the initial doping process identified in a similar dedoping experiment previously.[1] It is of course difficult to quantify the remaining dopant in the films for the same reasons as it is difficult to measure the TFSI concentration in doped films. However, in previous works doping with F4TCNQ at even 2.5 mol% led to much stronger $\pi - \pi^*$ bleaching than observed here.[15] This implies that the residual doping level must be much less than 2.5 mol%, corresponding to a concentration of about 2×10^{19} cm^{-3}. Note that the random error in the TFSI concentration measured by NMR, which is dominated by the signal to noise of the NMR measurement, corresponds to a concentration of $\pm 6 \times 10^{19}$ cm^{-3}—much larger than the upper bound on residual doping indicated by UV-vis. Therefore, incomplete dedoping does not appreciably contribute to error in these measurement.

These results of course rely on the assumption that there is no residual TFSI on the film surface, which would artificially inflate the measured carrier densities. To check for this possibility, the 100 s doped film we washed the film surface with CD_3CN prior to dedoping to verify the absence of residual ionic liquid on the surface. A small peak, slightly shifted (presumably due to a pH difference in the absence of TEA) is barely above the noise is visible in the ^{19}F spectra. We can imagine two possible origins of this soluble TFSI—(i) residual BMP TFSI on the film surface, or (ii) slight dedoping during CD_3CN exposure (e.g. by charge transfer from OH^- ions generated by adventitious water). To differentiate between these possibilities, we collected ^{1}H spectra of this sample, as well as DFBP and BMP TFSI standards. While several impurity peaks are visible in the ^{1}H spectra, we do not observe any signals corresponding to those in the BMP TFSI spectrum. Therefore, the weak TFSI signal observed in the washed sample must originate from slight dedoping during the washing step, and not residual BMP TFSI on the film surface. This result gives us further confidence in the carrier density measurements both from QNMR and XPS, as it validates our assumption that the number of TFSI ions measured equals the carrier density.

2.3 Calculation of volumetric carrier density

Both XPS and NMR provides a measure of molar ratio of anion to polymer. We can convert this to a volumetric carrier density if we know the mass density of the polymer. In undoped PBTTT the monomer density can be calculated as 9.07×10^{20} cm^{-3} from the reported density, 1.05 g/cm^3 [16] and the monomer molar mass. A value of 1.1 g/cm^3 [16] was used for the density of P3HT. We were unable to find reports of the density of DPP-BTz and IDTBT, therefore a value of 1 g/cm^3 was assumed. As with the conductivity values reported, we assume no volumetric change upon doping to allow for direct comparison of the differences in charge transport properties of the polymer.

Table S 2: Carrier densities measured by XPS and QNMR

		S 2p XPS		^{19}F QNMR		$FeCl_4^-$ UV-vis	
Polymer:Ion	Doping time	Molar ratio	N (10^{20} cm^{-3})	Molar ratio	N (10^{20} cm^{-3})	Molar ratio	N (10^{20} cm^{-3})
PBTTT:TFSI	100 s	0.915 ± 0.030	8.29 ± 0.30	0.822 ± 0.074	7.45 ± 0.67	–	–
PBTTT:TFSI	300 s	0.976 ± 0.026	8.84 ± 0.24	0.916 ± 0.071	8.31 ± 0.64	0.65 ± .022	5.89 ± 0.20
P3HT:HFSI	300 s	0.156 ± 0.008	6.45 ± 0.33	–	–	0.251 ± 0.003	10.38 ± 0.13
DPP-BTz:HFSI	300 s	0.571 ± 0.059	2.96 ± 0.31	–	–	0.414 ± 0.027	2.15 ± 0.14
IDTBT:HFSI	300 s	0.805 ± 0.063	3.73 ± 0.29	–	–	0.718 ± 0.048	4.00 ± 0.27

3 Ion exchange kinetics

The ion exchange kinetics of PBTTT:TFSI and P3HT:PF6 were reported previously.[1] In both cases, the system reaches maximum conductivity in about 60 seconds. We did not study the kinetics of DPP-BTz spectroscopically, however the electrical conductivity shows slightly faster kinetics than P3HT and PBTTT, reaching equilibrium in 20 - 30 seconds. The faster kinetics here would be consistent with phase segregation of dopant ions into amorphous domains, as discussed in Section 1.1. Further investigation will be necessary before any firm conclusions are possible, however.

IDTBT shows unique doping kinetics among the polymers studied in this work. Electrical conductivity in IDTBT is strongly dependent on doping exposure time (Figure S17), dropping nearly an order of magnitude below the peak conductivity at long doping times for some of the ions studied. As discussed in Section 1.1, the strongly time-dependent conductivity complicates our analysis of IDTBT to some degree. The timescale on which ion exchange occurs (Figure S4) is comparable to the time required to reach maximum conductivity, therefore some residual dopant remains in the film at the maximum conductivity point.

Nonetheless, we still observe a clear ionic size effect in IDTBT, visible in both Figure S17 and Figure 4f of the main paper. Additionally, the UV-vis spectra at maximum conductivity is nearly identical for PF_6, TFSI, HFSI, and PFSI (Figure S18a), indicating at maximum conductivity the doping level for each ion is very similar, and that the variation in conductivity with doping time derives from a strong carrier density dependence of the conductivity. Ions which require longer times to reach maximum conductivity also tend to show higher conductivity, therefore polymer degradation due to extended doping times does not seem to be significant on this timescale. Doping with $FeCl_4^-$ alone gives relatively low conductivity; IDTBT doped with $FeCl_3$ only (1 mM / 300s) gave a conductivity of 4.2 S/cm, lower than films with large ions which showed similar UV-vis spectra (c.f. BArF 600s, 11.1 S/cm; TFSI 30s, 5.9 S/cm) but somewhat higher than PF_6 at similar doping level (c.f. PF_6 30s, 3.0 S/cm). Therefore, the observed trend between conductivity and ionic size does not appear to be a side effect of the somewhat lower exchange efficiency in the maximum conductivity IDTBT samples.

The optimum doping level in IDTBT:BArF is somewhat lower, visible as the reduced P1 band intensity for BArF in Figure S18a, however several anecdotal observations suggest that higher conductivity may still be possible in IDTBT:BArF. Despite its large size, BArF is moderately hydrophilic and required drying before use in ion exchange,[17] as the residual water content limited the carrier density achievable with $FeCl_3$. Even after drying, the doping kinetics with Na BArF were much slower, suggestive of a reduced Fe^{3+} reduction potential due to residual water. If, on the other hand, ion exchange was simply slower in BArF due to slower ionic diffusion rates, we would instead expect to see fast initial doping followed by a slow increase in exchange efficiency. Long doping times with low concentrations of CAN or long electrochemical doping hold times strongly reduce conductivity, therefore it is not completely clear whether the drop in conductivity for IDTBT:BArF at 600s results from the same mechanism seen in the other ions.

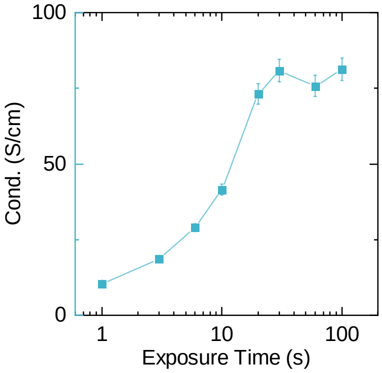

Figure S16: **DPP-BTz: doping time dependent conductivity** Doping solutions consisted of 100 /1 mM BMP TFSI / $FeCl_3$ in AN

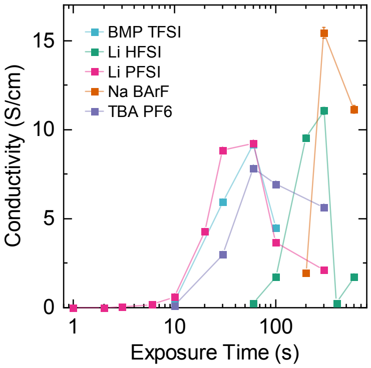

Figure S17: **IDTBT: doping time dependent conductivity** Doping solutions consisted of 100 /1 mM electrolyte / $FeCl_3$ in AN. Corresponding UV-vis spectra are shown in Figure S18

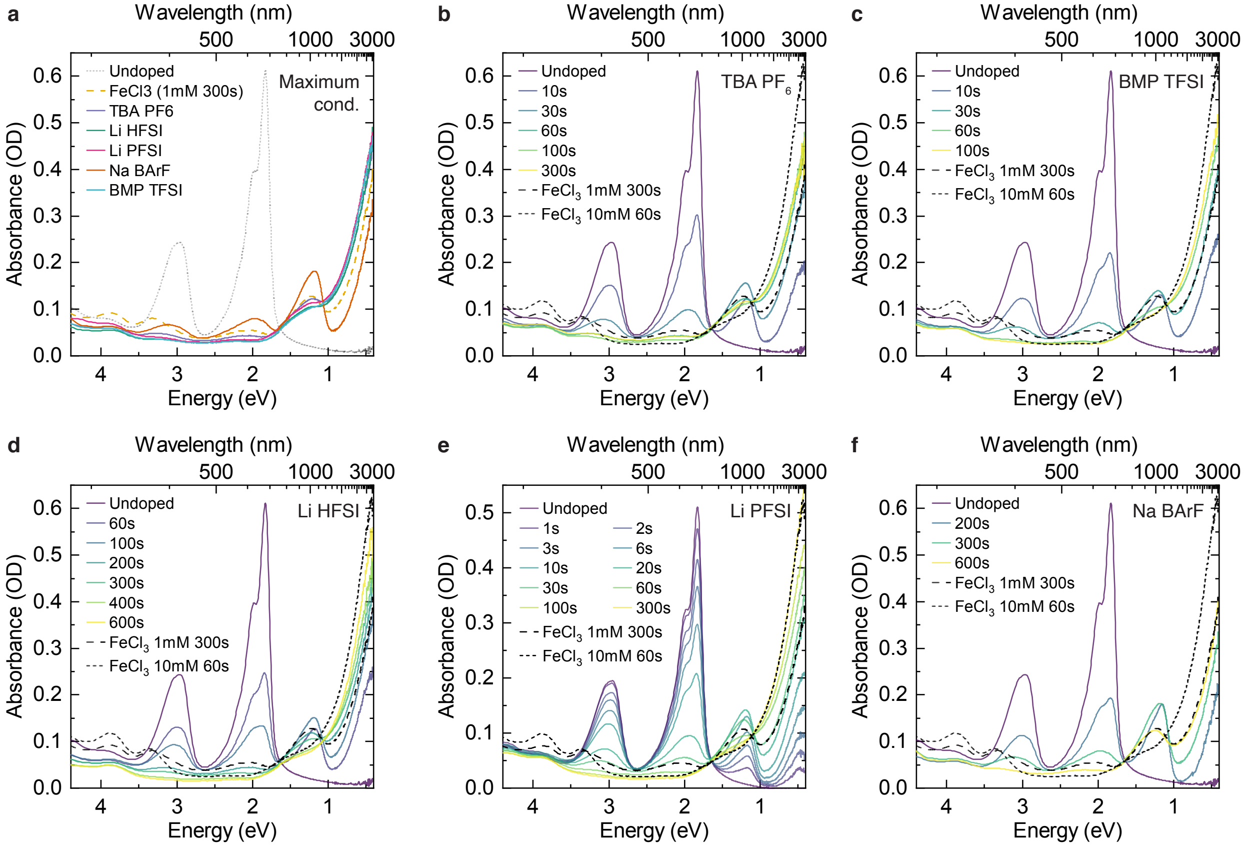

Figure S18: **IDTBT doping time dependent UV-vis** Doping solutions consisted of 100 /1 mM electrolyte / $FeCl_3$ in AN. a) Spectra of films at the maximum conductivity point for each ion studied. b-f) Spectra of films doped for varying times; electrolyte is indicated in the upper right corner of each plot.

4 Full GIWAXS results

Full GIWAXS results for each sample are given below. Details of the GIWAXS data processing are given in the methods section, main paper.

4.1 PBTTT

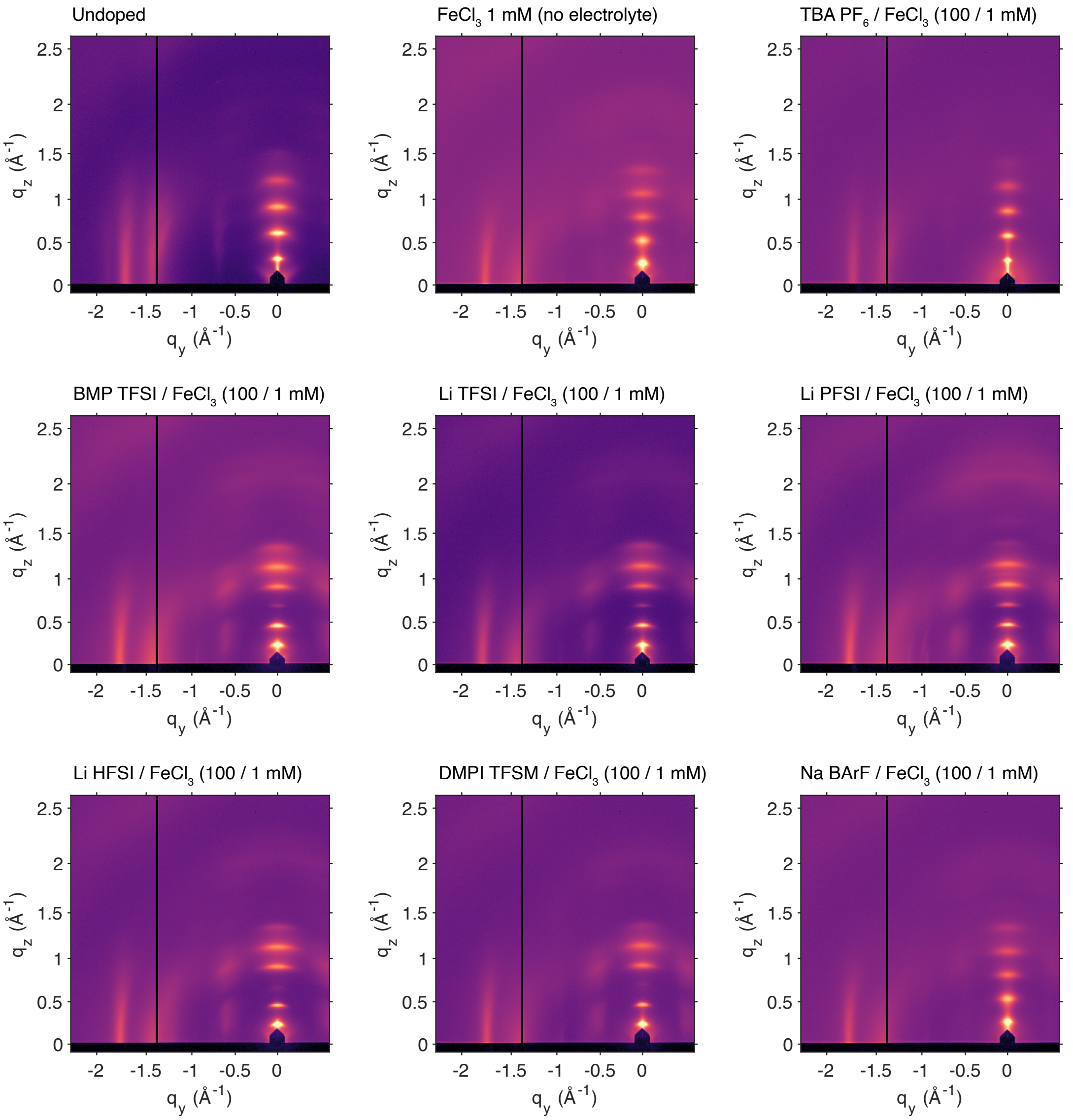

Figure S19: **PBTTT ion dependent GIWAXS patterns, 5 min. doping time (max. doping level)**. Doping solutions contained 1 mM $FeCl_3$ and 100 mM electrolyte (see label above each plot).

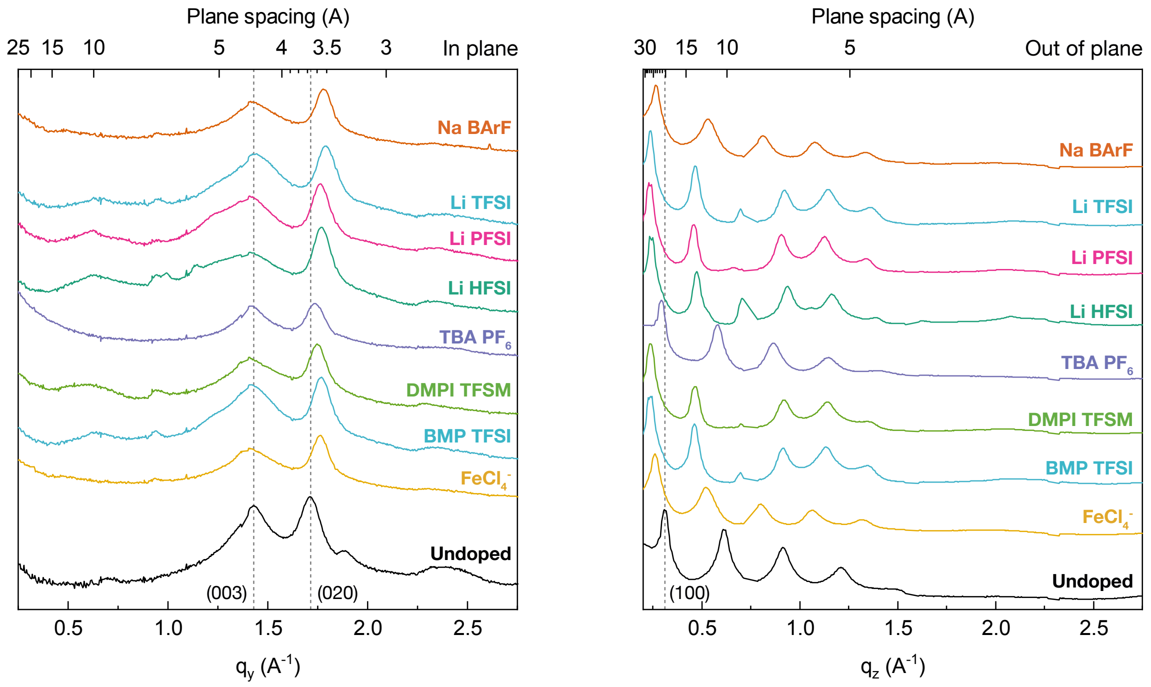

Figure S20: **PBTTT ion dependent GIWAXS linecuts, 5 min. doping time (max. doping level)**. Doping solutions contained 1 mM $FeCl_3$ and 100 mM electrolyte (see label above each plot).

Table S 3: Lattice spacings and paracrystallinity extracted from PBTTT GIWAXS data

Electrolyte	q_{lam}	d_{lam}	$q_{\pi-\pi}$	$d_{\pi-\pi}$	$g_{\pi-\pi}$
Undoped	0.310	20.24	1.712	3.67	9.41
None ($FeCl_4^-$)	0.260	24.13	1.762	3.57	8.20
BMP TFSI	0.237	26.54	1.767	3.56	8.44
DMPI TFSM	0.237	26.47	1.748	3.59	8.54
TBA PF6	0.293	21.45	1.737	3.62	8.98
Li HFSI	0.241	26.09	1.768	3.55	8.16
Li PFSI	0.234	26.89	1.764	3.56	8.39
Li TFSI	0.237	26.54	1.791	3.51	8.65
Na BArF	0.265	23.71	1.779	3.53	8.32

4.2 P3HT

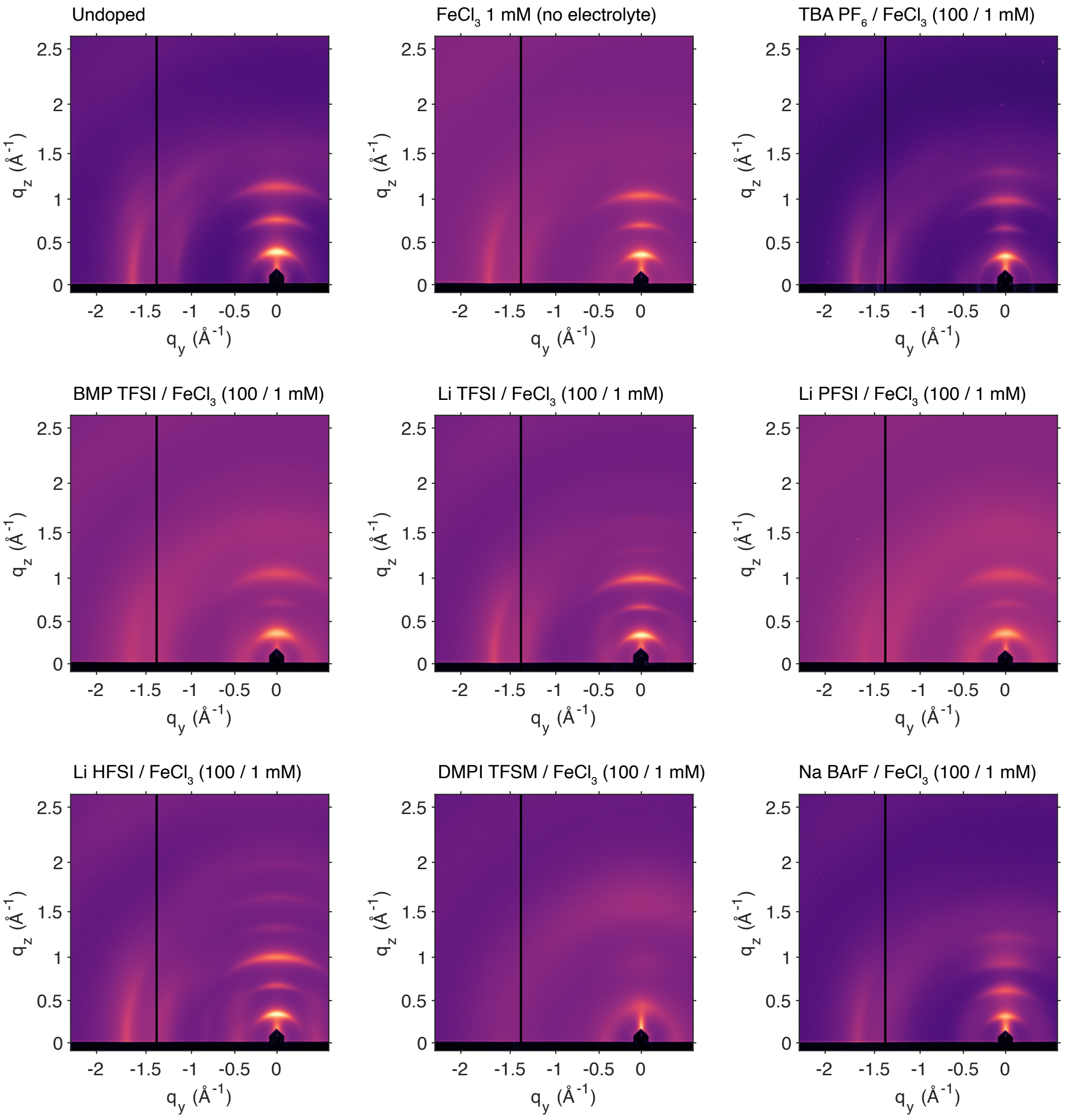

Figure S21: **P3HT ion dependent GIWAXS patterns, 5 min. doping time (max. doping level)**. Doping solutions contained 1 mM $FeCl_3$ and 100 mM electrolyte (see label above each plot).

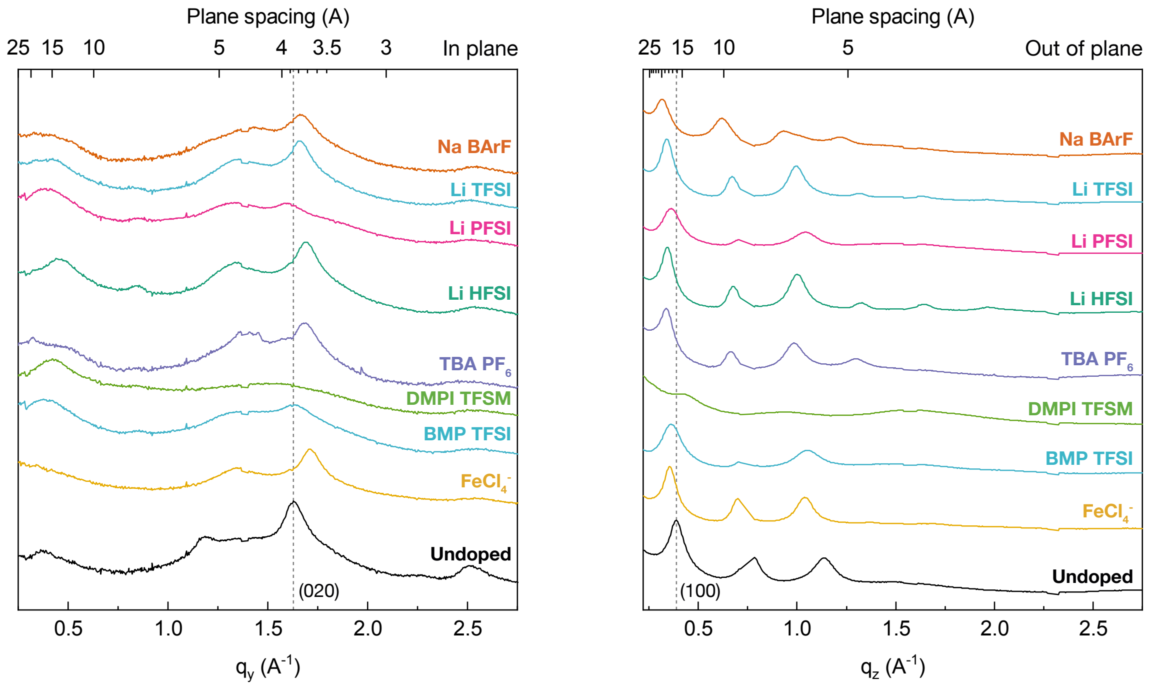

Figure S22: **P3HT ion dependent GIWAXS linecuts, 5 min. doping time (max. doping level)**. Doping solutions contained 1 mM $FeCl_3$ and 100 mM electrolyte (see label above each plot).

Table S 4: Lattice spacings and paracrystallinity extracted from P3HT GIWAXS data

Electrolyte	q_{lam}	d_{lam}	$q_{\pi-\pi}$	$d_{\pi-\pi}$	$g_{\pi-\pi}$
Undoped	0.388	16.21	1.631	3.85	10.76
None ($FeCl_4^-$)	0.355	17.72	1.711	3.67	10.49
BMP TFSI	0.362	17.36	1.627	3.86	14.73
DMPI TFSM	0.421	14.92	1.538	4.08	23.12
TBA PF6	0.336	18.72	1.683	3.73	10.54
Li HFSI	0.342	18.37	1.692	3.71	11.08
Li PFSI	0.361	17.41	1.593	3.94	14.28
Li TFSI	0.339	18.53	1.660	3.79	11.24
Na BArF	0.314	20.03	1.666	3.77	12.22

4.3 DPP-BTz

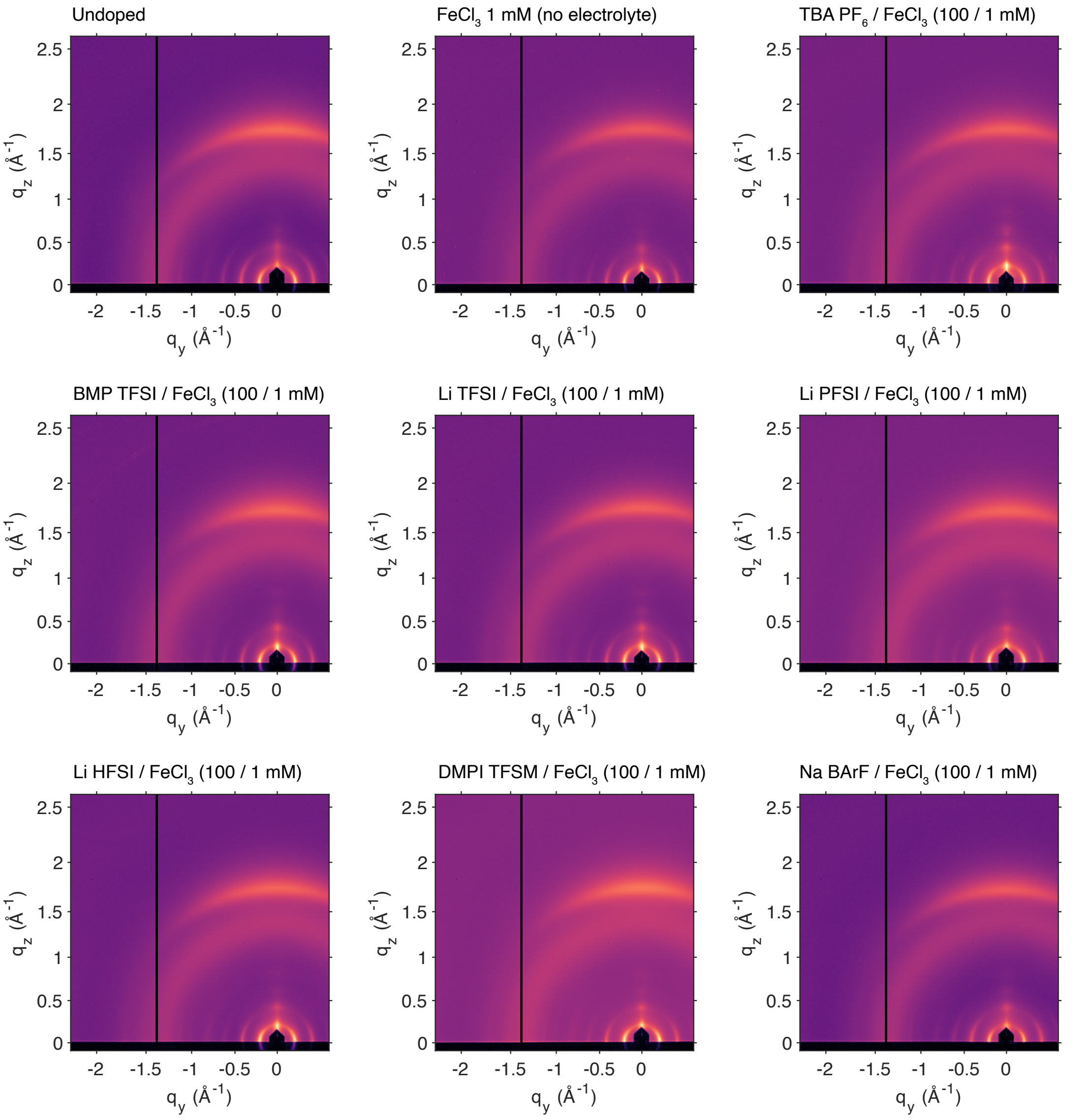

Figure S 23: **DPP-BTz ion dependent GIWAXS patterns, 5 min. doping time (max. doping level)**. Doping solutions contained 1 mM $FeCl_3$ and 100 mM electrolyte (see label above each plot).

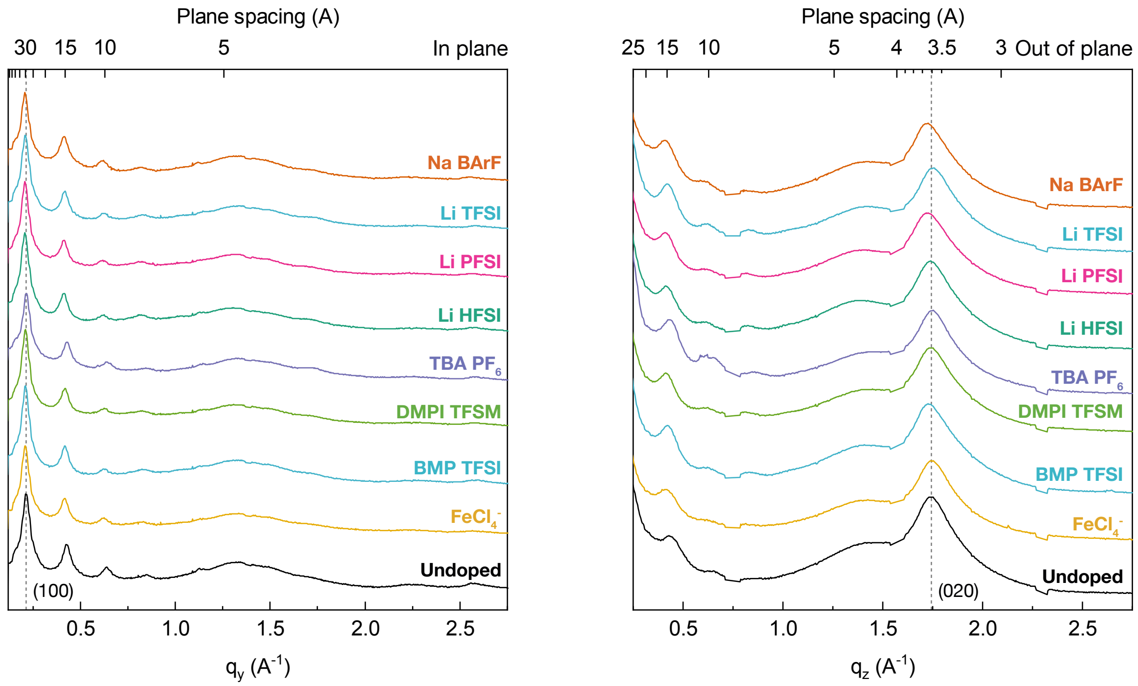

Figure S24: **DPP-BTz ion dependent GIWAXS linecuts, 5 min. doping time (max. doping level)**. Doping solutions contained 1 mM $FeCl_3$ and 100 mM electrolyte (see label above each plot).

Table S 5: Lattice spacings and paracrystallinity extracted from DPP-BTz GIWAXS data

Electrolyte	q_{lam}	d_{lam}	$q_{\pi-\pi}$	$d_{\pi-\pi}$	$g_{\pi-\pi}$
Undoped	0.215	29.22	1.743	3.60	11.92
None ($FeCl_4^-$)	0.210	29.95	1.749	3.59	12.31
BMP TFSI	0.211	29.78	1.737	3.62	12.64
DMPI TFSM	0.211	29.77	1.745	3.60	12.83
TBA PF6	0.216	29.09	1.750	3.59	12.22
Li HFSI	0.208	30.20	1.743	3.60	12.44
Li PFSI	0.209	30.12	1.729	3.63	13.33
Li TFSI	0.210	29.86	1.756	3.58	12.45
Na BArF	0.208	30.15	1.728	3.64	12.51

4.4 IDTBT

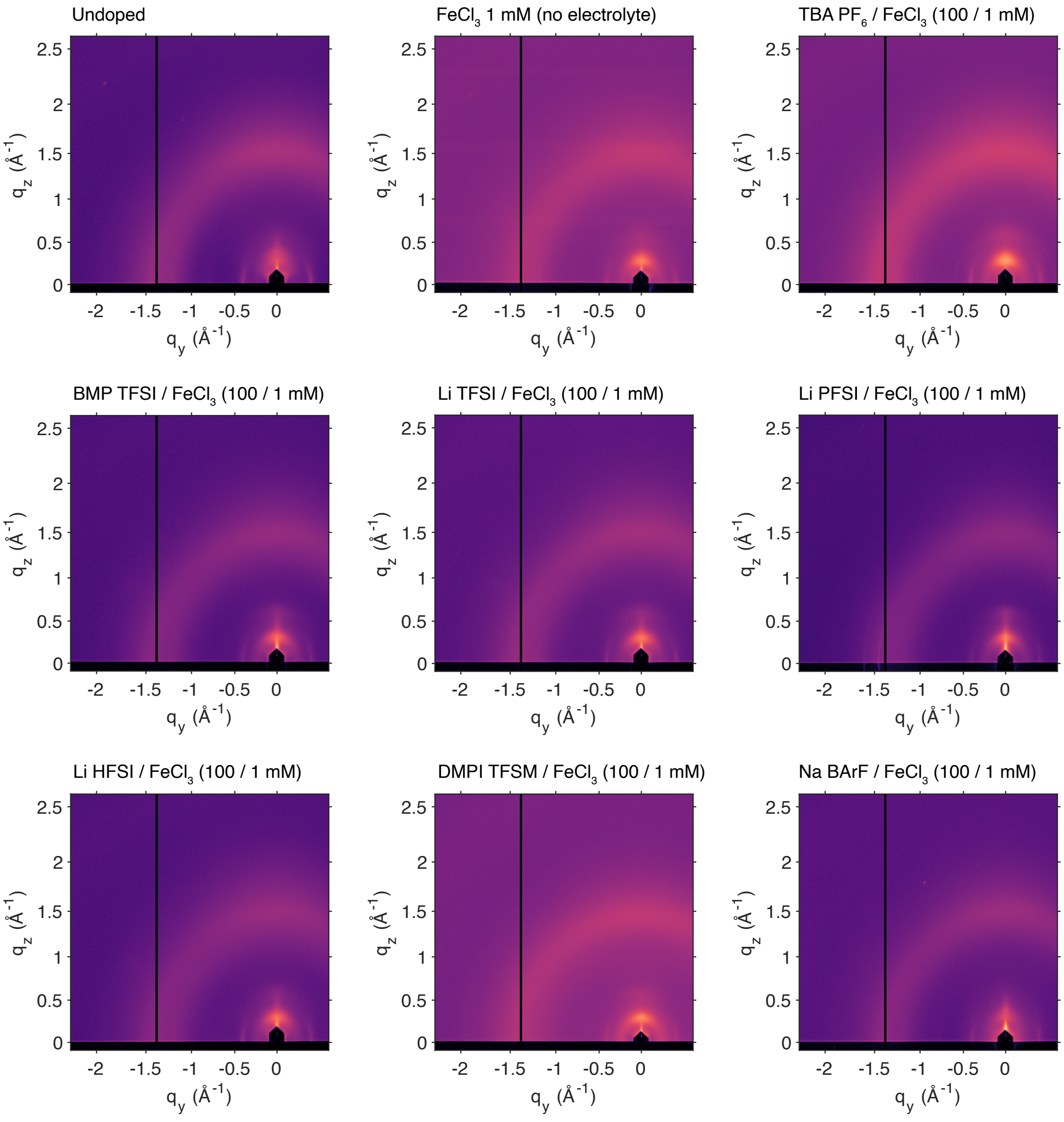

Figure S25: **IDTBT ion dependent GIWAXS patterns, 5 min. doping time (max. doping level)**. Doping solutions contained 1 mM $FeCl_3$ and 100 mM electrolyte (see label above each plot).

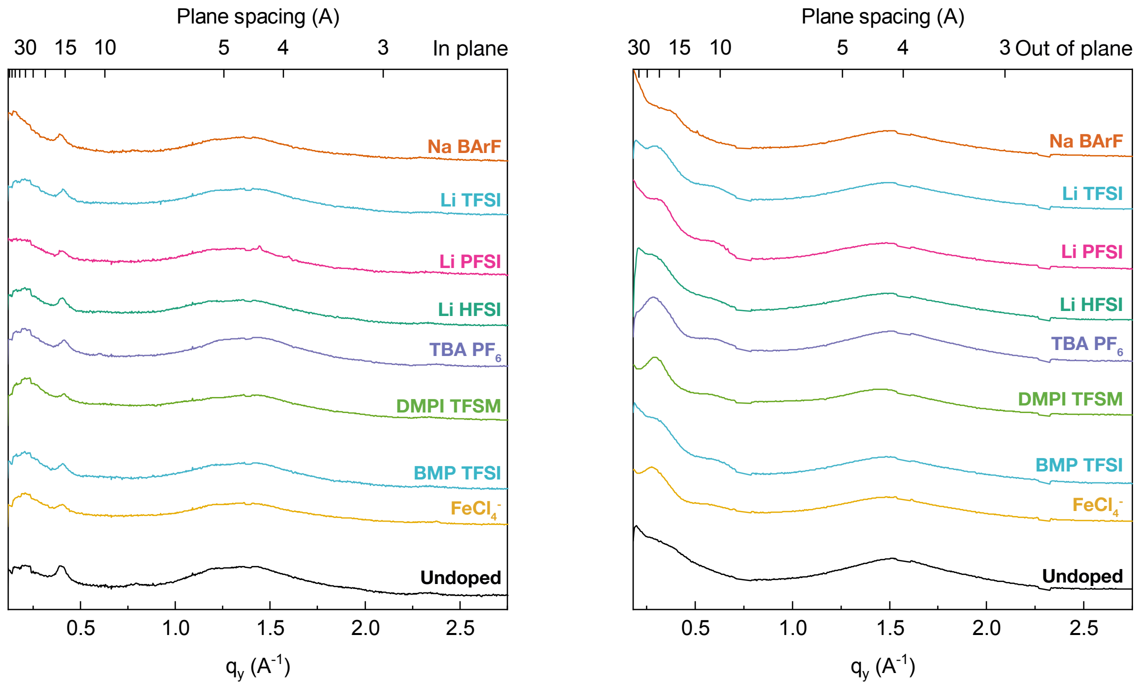

Figure S26: **IDTBT ion dependent GIWAXS linecuts, 5 min. doping time (max. doping level)**. Doping solutions contained 1 mM $FeCl_3$ and 100 mM electrolyte (see label above each plot).

Table S 6: Lattice spacings and paracrystallinity extracted from IDTBT GIWAXS data

Electrolyte	$q_{\pi-\pi}$	$d_{\pi-\pi}$	$g_{\pi-\pi}$
Undoped	1.509	4.16	24.23
None ($FeCl_4^-$)	1.476	4.26	25.89
BMP TFSI	1.474	4.26	23.65
DMPI TFSM	1.436	4.38	24.61
TBA PF6	1.507	4.17	24.43
Li HFSI	1.471	4.27	27.28
Li PFSI	1.461	4.30	24.88
Li TFSI	1.484	4.23	25.84
Na BArF	1.491	4.21	25.85

5 Full dataset correlation with g vs. λ_x

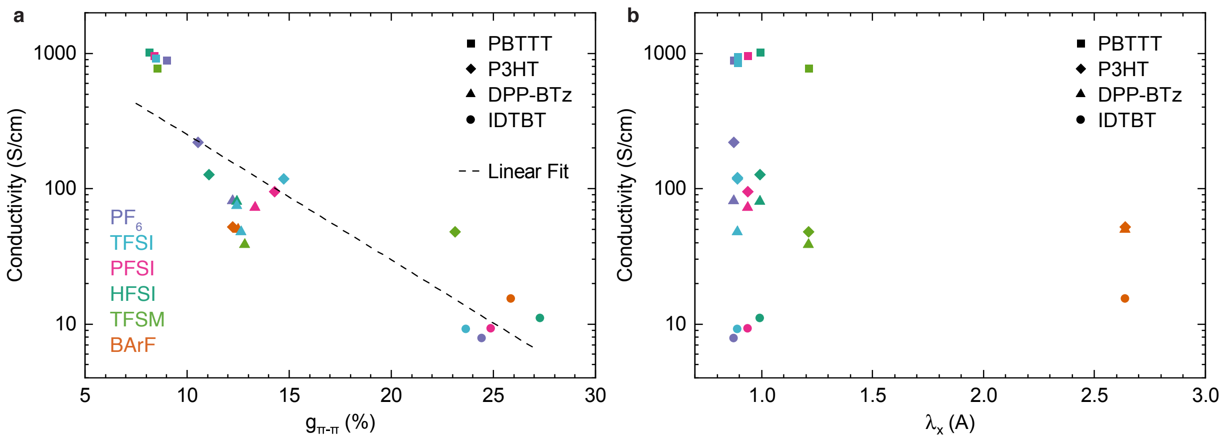

Figure S27: Plots of electrical conductivity vs. a) paracrystallinity (g), and b) ionic size, defined as the smallest component of the ionic gyration tensor, λ_x.

We briefly discuss the correlation of electrical conductivity with paracrystallinity $g_{\pi-\pi}$, and ionic size, measured as the smallest component of the ionic gyration tensor λ_x. As discussed in the main text, we observe a surprisingly strong correlation between $g_{\pi-\pi}$ and conductivity in our full dataset (Figure 5, main paper, reproduced in Figure S27a). The strength of this correlation across the full dataset suggests that variations in FET mobility are less critical in this highly doped regime, presumably due to increased backbone planarity resulting from the quinodal electronic structure characteristic of polarons. In comparison, a plot of the full dataset vs. λ_x (Figure S27b) reveals no correlation whatsoever. If free carrier generation was inefficient (i.e. ionic trapping is significant) we would instead expect to instead see a strong correlation with λ_x due to reduced trapping from larger ions. As noted in Figure 4, we do not observe a positive correlation between σ and λ_x even within individual polymer datasets, except in the case of IDTBT. The complete absence of any relationship between λ_x and conductivity at this high doping level provides clear evidence that ionic trapping does not limit conductivity at these high carrier densities.

6 General model for doped conjugated polymers

The electronic and charge transport properties of highly-doped paracrystalline lamellae are described with a model for spinless holes on a 2D disordered lattice with Coulomb interactions among holes, and between holes and dopant anions, the latter being described as classical charges. The sites of our model correspond to monomer or co-monomers (conjugated polymer units separated by soft torsions, e.g. thiophene or bi-thiophene in PBTTT), to each of which is associated a site orbital (monomer HOMO, ϕ_i). Charge neutrality in the system determines the band filling, i.e. the number of holes on polymer sites equals the number of dopants, placed at distance R_{dop} from the lamella plane. The system Hamiltonian reads

$$H = \sum_{\langle i,j \rangle} t_{ij} \left(c_i^\dagger c_j + h.c. \right) + \sum_i V_i^{(ion)} \, \hat{n}_i + \frac{1}{2} \sum_{i,j} V_{ij} \, \hat{n}_i \hat{n}_j \tag{4}$$

where $c_i^\dagger$ (c_i) creates (annihilates) a spinless hole at site i, $\hat{n}_i = c_i^\dagger c_i$, and t_{ij} are charge transfer integrals. The potential of ions is $V_i^{(ion)} = -\sum_k V_{ki}$, with k running on ion sites, and $V_{ij} = e^2/(\varepsilon_r |\mathbf{r}_j - \mathbf{r}_i|)$ being the Coulomb interaction, screened by the medium dielectric constant – e is the elementary charge. The value $\varepsilon_r = 3.5$ is used throughout this work. The adoption of spinless particles implicitly neglects the double charging of polymer sites, similar to the large Hubbard's U limit of a spinful model. Hamiltonian (4) represents the natural extension to the case of multiple dopants of the model proposed in Ref. 18, with the main difference that here the full ionization of dopants is implicitly assumed (no hybridization). This is well justified in the case of ion-exchange doping, since the dopant ions are charge-neutralization agents and cannot lead to the formation of charge-transfer complexes.

Because of the exponential growth of the Hilbert space with the system size, the exact diagonalization of Hamiltonian (4) is computationally prohibitive for the systems of interest in the present study. We solve this problem within the mean field (Hartree-Fock) approximation, leading to the effective one-particle Hamiltonian

$$\hat{F}[P] = \sum_{\langle i,j \rangle} t_{ij} \left(c_i^\dagger c_j + h.c. \right) + \sum_i \left(V_i^{(ion)} + V_i^H[P] \right) \hat{n}_i \tag{5}$$

where V_i^H is the Hartree potential at site i, accounting for Coulomb and exchange interactions, which depends self-consistently on the density matrix P. This self-consistent problem is solved with standard iterative approaches (solution mixing), up to convergence of the total energy.

Periodic boundary conditions are applied along the x and z directions, in order to minimize size effects of finite lattices. Site potentials, $(V_i^{(ion)} + V_i^H)$ have been evaluated with direct electrostatic sums. The convergence of these long-range interactions does not pose particular problems in 2D, provided that dopant ions are placed on both sides of the polymer plane.

The densities of states (DOS) have been calculated from the eigenvalues of Hamiltonian (5) for hole states, referred to a common Fermi level (E_F), and reversed in sign as convenient for an electron representation. DOS and observables have been averaged over numerical results for 10 systems, corresponding to different random realizations of the structural and energetic disorder. The eigenstates of Hamiltonian (5) are expressed as linear combinations of site orbitals,

$$\psi_n = \sum_i c_{in} \, \phi_i, \tag{6}$$

that are assumed to be orthogonal and normalized. The spread of the hole wavefunctions, i.e. the number of sites over which the particle is delocalized, is quantified by the participation ratio

$$L_n = \frac{1}{\sum_i |c_{in}|^4}. \tag{7}$$

We further distinguish between the delocalization along a polymer chain and among different chains. The inter-chain spread is defined as

$$L_n^{inter} = \frac{1}{\sum_l p_{nl}^2} \tag{8}$$

where $p_{nl} = \sum_{i}^{i\in l} |c_{in}|^2$ is the wavefunction amplitude on chain l. The average intra-chain spread is then $L_n^{intra} = L_n / L_n^{inter}$.

The dc conductivity has been calculated within the framework of the transient localization theory.[19,20] The starting point is the Kubo-Greenwood expression for the optical conductivity:

$$\tilde{\sigma}_\alpha(\omega) = \frac{\pi}{v\hbar} \frac{\left(1 - e^{-\beta\hbar\omega}\right)}{\hbar\omega} \sum_{mn}{}' f_m(1 - f_n) \, |\langle n|\hat{J}_\alpha|m\rangle|^2 \, \delta(\hbar\omega - E_n + E_m) \tag{9}$$

where α labels Cartesian components, $\hbar$ is the reduced Planck constant, v the system volume, $\beta = 1/(k_B T)$, k_B is the Boltzmann constant, T the absolute temperature, f the Fermi function, δ the Dirac delta function, and $|m\rangle$ is the eigenstate of Hamiltonian (5) of energy E_m. The current operator reads

$$\hat{J}_\alpha = -\iota e \sum_{\langle i,j \rangle} (\mathbf{r}_{ij} \cdot \hat{\kappa}_\alpha) \, t_{ij} \, (c_i^\dagger c_j - h.c.) \tag{10}$$

where ι is the imaginary unit, e the elementary charge, $\mathbf{r}_{ij} = \mathbf{r}_j - \mathbf{r}_i$ the inter-site distance vector and $\hat{\kappa}_\alpha$ the versor of the Cartesian axis α.

The Kubo-Greenwood static ($\omega = 0$) conductivity is strictly zero for a two-dimensional system with charges localized by a static energetic disorder. The disorder is, however, never static in soft organic materials, in which it gets modulated by low-frequency thermal lattice vibrations enabling a finite diffusivity to charge carriers. The relaxation time approximation provides an effective framework to account for these transient localization phenomena.[19,20] In this framework, the optical conductivity can be obtained as a convolution product of $\tilde{\sigma}(\omega)$ in Eq. (9) with a zero-centered Lorentzian function (Λ) with half-width at half-maximum γ, which corresponds to the typical vibrational energy of the lattice. The dc conductivity hence reads

$$\sigma_\alpha = \frac{\pi}{\hbar v} \sum_{mn}{}' (1 - f_m)(1 - f_n) \, \frac{e^{-\beta\epsilon_m} - e^{-\beta\epsilon_n}}{E_n - E_m} \, |\langle n|\hat{J}_\alpha|m\rangle|^2 \, \Lambda(E_n - E_m; 0, \gamma) \tag{11}$$

where $\epsilon_m = E_m - E_F$. Conductivity values have been averaged over 100 realization of the disorder. Unless specified otherwise, we present results obtained for $\gamma = 5$ meV. Other plausible choices for this parameter do not qualitatively affect the results, see Figure 28.

6.1 Model parameterization and supplementary results

The model considers a disordered rectangular lattice, sketched in Figure 6a of the manuscript with dopant ions placed at distance R_{dop} from the polymer plane. Ions are arranged in a face-centered rectangular lattice commensurate with the simulation cell. The system geometry is chosen in such a way that ions form a slightly deformed hexagonal lattice, which would correspond to the minimum-energy configuration for this system of charges. We have explicitly verified that our results do minimally depend on the specific choice of the ions lattice, e.g. introducing random disorder in the ions positions and by considering other lattices.

We present results for one-third band filling, fixed by the experimental estimate for the dopants density in PBTTT given in Section 2, $\rho = 33.3\%$, i.e. one dopant per monomer (3 co-monomers). These results are representative of the highly-doped regime attained in our experiments, see Figure 29. These data demonstrate that, at high doping loads, the specific value of ρ has a negligible effect on the electronic states at the Fermi and hence on charge transport.

Calculation results have been obtained for a lattice of 48×14 sites, unless specified otherwise. This system is sufficiently large to converge the DOS and the properties of the states around the Fermi level (E_F), although residual size effects can be detected in the low-disorder limit, see Figure 37. Calculations for systems with longer polymer chains allowed to ascribe the oscillations in the middle of the DOS to the limited system size. These artifacts are nevertheless irrelevant for the states around E_F and for transport.

The polymer lattice has regular spacing a along the x direction, corresponding to the polymer backbone axis. We fixed a =4.5 Å, as relevant to PBTTT. The lattice spacings along the π-stacking direction z are random numbers drawn from a skewed Reinhold distribution[21,22] whose parameters depend on $g_{\pi-\pi}$ (see

below). Site positions are then determined from random lattice spacings in a cumulative fashion, realizing a 1D paracrystalline model lattice along z. The distance between lamellae, relevant only to determine v in Eqs. 9,11, is set to 25 Å.

The adoption of a skewed distribution for lattice spacings [23] is a necessary element to account for the increase in the mean π-stacking direction with $g_{\pi-\pi}$ observed in our samples. Diffraction data collected on the full set of polymers and dopants considered in this study (see Figure 30) allowed us to establish the following linear empirical relationship between $d_{\pi-\pi}$ and paracrystallinity

$$d_{\pi-\pi} = 3.235 + 3.879\, g_{\pi-\pi}, \tag{12}$$

which has been used throughout this work to build the paracrystalline lattice, as well as the model for energetic disorder on polymer chains, to the single parameter $g_{\pi-\pi}$. The specific choice to opt for the Reinhold function (see Figure 30) is motivated by its use in previous studies of paracrystalline polymers, [21,22] and by the fact that it is straightforward to obtain skewed distributions with predefined mean value and variance. We expect, however, that other skewed distributions with same mean value and variance would provide similar results.

The model accounts for nearest-neighbor charge hopping between monomer units along the x and z direction, as well as for the associated non-local energetic disorder associated to the low-frequency lattice motion. The distribution of charge-transfer integrals in the presence of paracrystalline disorder has been entirely parameterized from atomistic calculations on PBTTT. We expect that the order of magnitude of hopping interactions and their fluctuations, are broadly representative also of the other polymers considered in this study.

Inter-chain and intra-chain transfer integrals between thiophene (T) and bithiophene (TT) fragments have been computed at the DFT level (PBE0/def2-SVP, ORCA code [24]). These calculations were performed for a crystalline structure of PBTTT obtained from force-field simulations [25,26] and then optimized with periodic DFT calculations (PBE-D3, CASTEP code [27]). Starting from this equilibrium structure, transfer integrals have been computed as a function of the inter-chain π stacking distance and of the inter-ring torsional angle, as shown in Figure 31. At the equilibrium structure, intra-chain transfer integrals are one order of manitude larger that inter chain ones ($\sim$1 eV vs. $\sim$0.1 eV). The energetic disorder on inter-chain hopping follows directly from the distribution of stacking distances, as illustrated in Figure 32.

Coming to intra-chain energetic disorder, we assume that this is determined by torsional disorder along the polymer backbone, which is known to be the leading contribution in conjugated polymers characterized by soft torsions. [25] A set of molecular dynamics simulations (MD, Materials studio package) employing a force field with re-parameterized torsions [25,26], has been performed on a PBTTT crystalline samples to quantify the torsional disorder. Room-temperature NVT simulations have been performed at the equilibrium volume, and for a simulation cell expanded by 10 and 20% along the π-stacking direction, in order to mimic the effect of paracrystallinity on d_π (see Fig. 30). Results in Figure 33 reveal an increase in torsional disorder, here quantified by the standard deviation of the absolute value of the torsional angle (δ_ϕ), with the π-stacking distance, corresponding to a larger backbone flexibility in loosely packed chains. We also note that the most probable conformation for TT-T torsions at large d_π (+20%) is not the planar one, similar to an isolated chain. [26]

To finally account for the $g_{\pi-\pi}$-dependent effect of torsional disorder on intra-chain hoppings, we have proceeded as follows: (i) we have averaged δ_ϕ over TT-T and T-T torsions as the model makes no distinction between the two co-monomers; (ii) We have performed a linear fit of δ_ϕ vs. $d_{\pi-\pi}$ (and hence vs. $g_{\pi-\pi}$, using Eq. 12), obtaining the relationship between torsional disorder and paracrystallinity: $\sigma_\phi = 6.9 + 117.8\, g_{\pi-\pi}$; (iii) We have assumed a Gaussian distribution of torsional angles with standard deviation $\sigma_\phi(g_{\pi-\pi})$; (iv) We have related intra-chain transfer integrals to torsional angles according to the DFT data in data in Figure 31. This procedure and the resulting distributions of intra-chain hoppings as a function of $g_{\pi-\pi}$ are illustrated in Figure 34.

The effect of high-frequency (quantum) vibrations is taken into account through an effective renormalization of hopping integrals: $t_{ij} \rightarrow \tilde{t}_{ij} = t_{ij} e^{-S}$. [28] $S = \lambda_e/\hbar\omega_e$ is the Huang-Rhys factor of an effective vibrational mode with frequency ω_e and relaxation energy λ_e. The relaxation energy associated to high-frequency modes in oligothiophenes [29] is similar to the typical C-C stretching energies in conjugated systems.

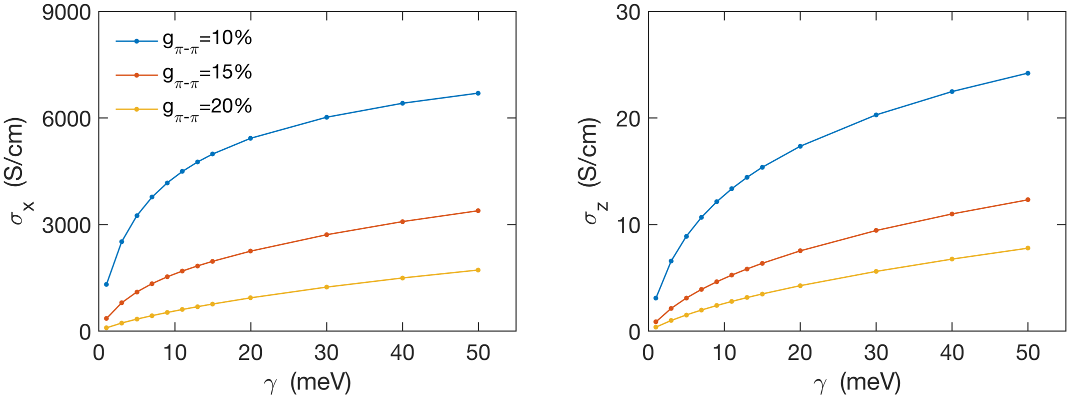

Figure S28: Dependence of the dc conductivity on the characteristic energy of low-frequency lattice vibrations, γ. The different curves show the conductivity for different values of the paracrystallinity parameter $g_{\pi-\pi}$31.

We hence mostly present results obtained for $S = 1$, the effect of varying S in a plausible range is addressed in Figure 36. Such a band renormalization effect is an important one, as it affects the competition between charge delocalization and Coulomb interactions in a disordered medium. Indeed, large S values determine narrow bands favoring localized states that are more sensitive to energetic disorder and Coulomb interactions. On the other hand, a reduction of S suppresses the Coulomb gap at E_F, pushing the system toward a metallic state.

An overview of the mean values and fluctuation of the parameters of the model Hamiltonian (5), as function of the paracrystallinity and R_{dop} parameters, is given in Figure 35. Complementary calculation results, characterizing the effect of paracrystallinity-induced disorder on the electronic structure of doped polymers, are shown in Figure 38 and Figure 39.

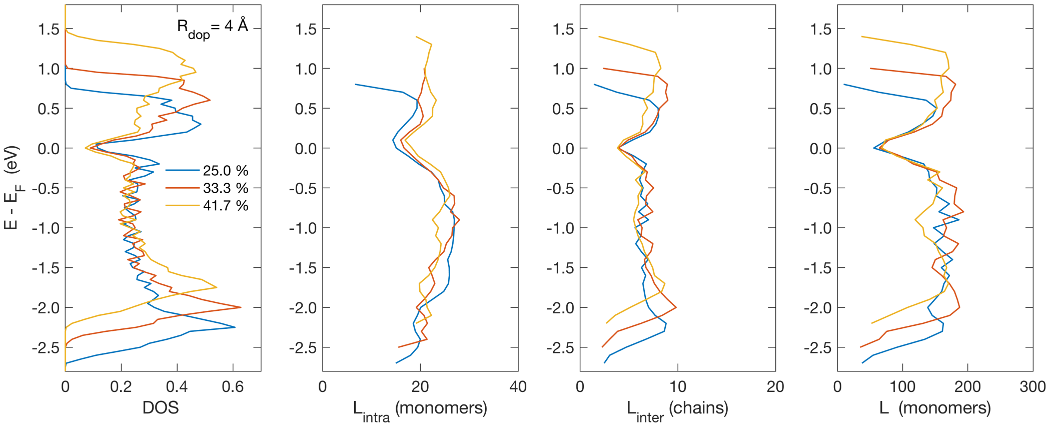

Figure S29: Electronic properties as a function of the dopants density (see legend). Panels from left to right show the DOS and charge delocalization observables (L_{intra}, L_{inter}, L). The latter are presented as eigenstates expectation values, averaged over energy intervals, as a function of the bin center. The Coulomb gap, as well as the pronounced localization of states at E_F, are general features of the model for highly-doped polymers, weakly dependent on the specific doping load. Results obtained for $g_{\pi-\pi}$=10 % and $R_{\rm dop}$=4 Å.

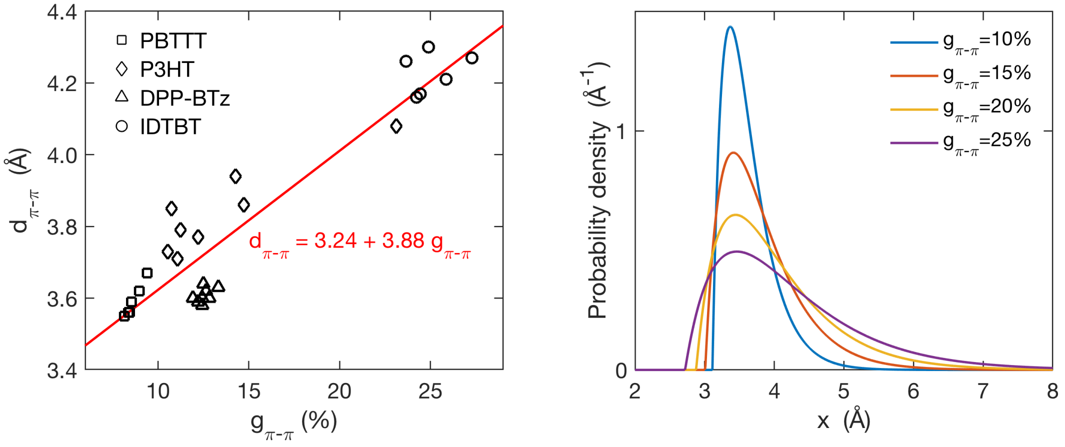

Figure S30: Left: Experimental relationship between paracrystallinity and the π stacking distance, revealing an approximately linear trend. Right: Reinhold distribution of π stacking distances for different $g_{\pi-\pi}$ values.

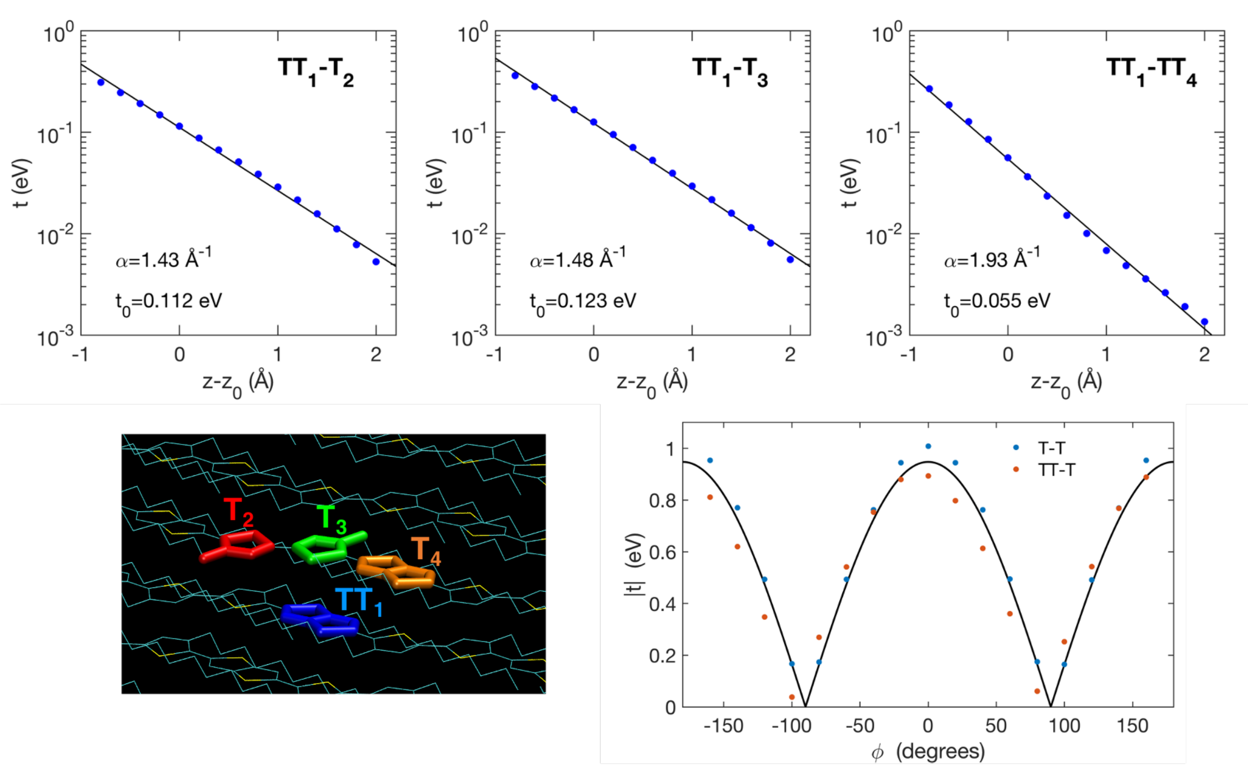

Figure S31: Transfer integrals between thiophene (T) and bithiophene (TT) fragments in crystalline PBTTT as a function of the inter chain distance (top, z_0=3.50 Å) and intra-chain torsional angle ϕ (bottom-right). Calculations performed at the PBE0/def2-SVP level. The z dependence of transfer integrals has been fitted with an exponential function $t = t_0 e^{-\alpha(z-z_0)}$, with parameters noted in each panel. The torsional dependence of the hopping is approximated as $t(\phi) = c\sqrt{1+\cos(2\phi)}$, with $c = 0.67$ eV.

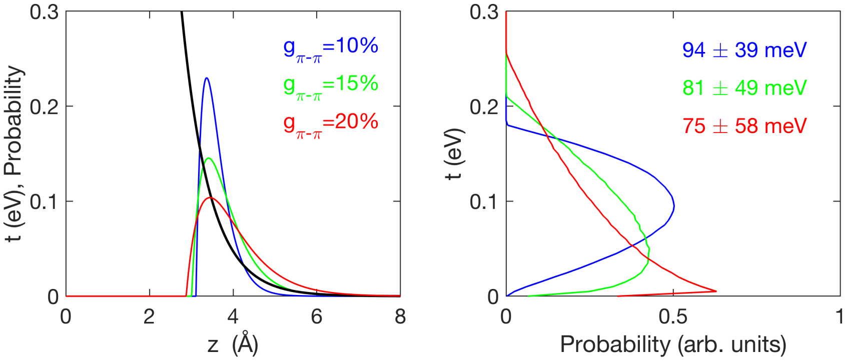

Figure S32: Left: Distributions of inter-chain distances for different values of $g_{\pi-\pi}$, superimposed to the transfer integral dependence on the π-stacking distance, $t(z) = t_0 e^{-\alpha(z-z_0)}$(black line). The values t_0=0.1 eV, α=1.5 Å^{-1} and z_0=3.5 Å have been used throughout this study (see Figure 32). Right: Distributions of inter-chain transfer integrals for different $g_{\pi-\pi}$. Mean values and standard deviations are annotated.

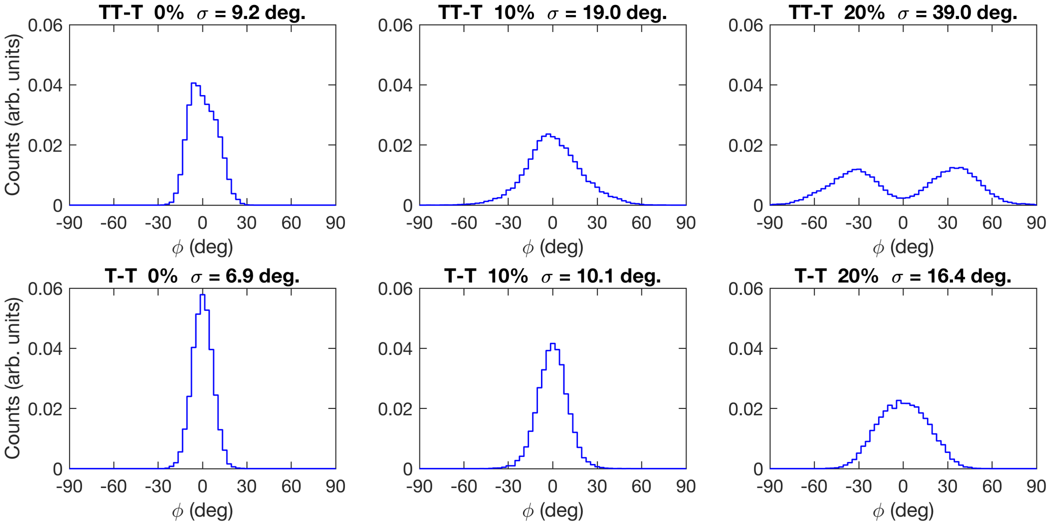

Figure S33: Distributions of intra-chain TT-T (top) and TT-T (bottom) torsional angles obtained from a set of room-temperature NVT molecular dynamics simulations, in which the means spacing between chains has been expanded form the equilibrium value (3.46 Å, 0%, left) by 10% (middle) and 20% (right).

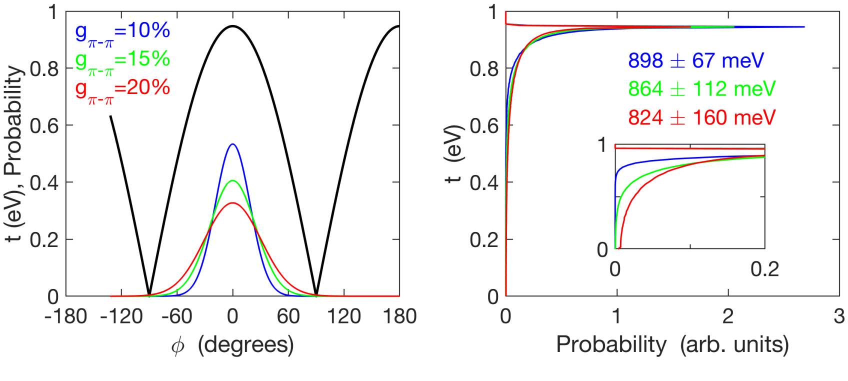

Figure S34: Left: Transfer integral dependence on the torsional angle ϕ (black line, see Figure 31), superimposed to the distributions of ϕ for different values of $g_{\pi-\pi}$. Right: Corresponding distributions of intra-chain transfer integrals. Mean values and standard deviations are annotated.

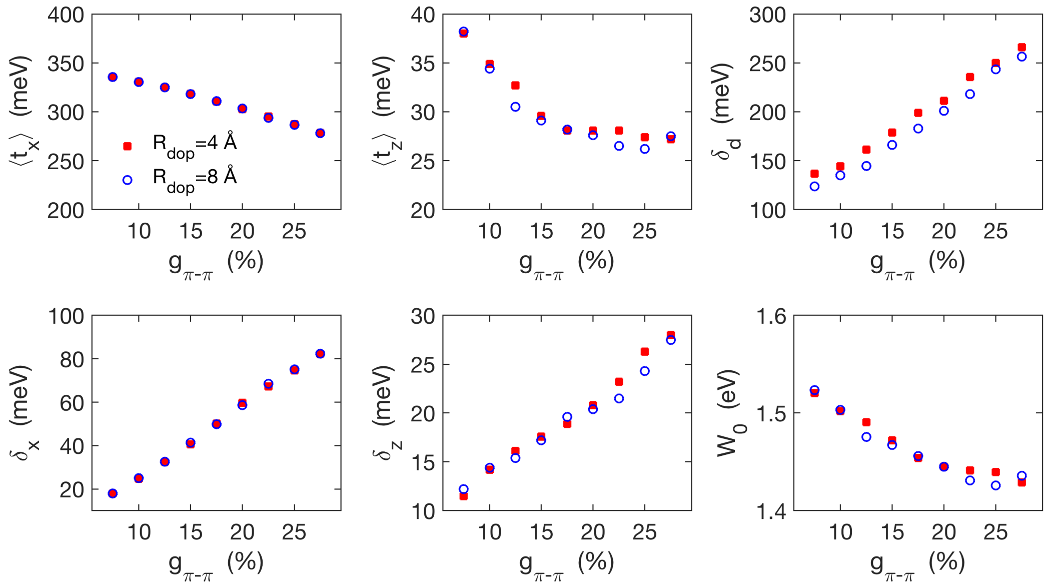

Figure S35: Evolution of the model parameters with paracrystallinity. The different panels show the mean value ($\langle t \rangle$) and standard deviation (δ) of intra-chain (x) and inter-chain (z) charge transfer integrals, the standard deviation of site potentials (diagonal disorder, δ_d), and the bandwidth (W_0) of the associated, Coulomb interactions-free, tight-binding model. The main effect of paracrystallinity is a large increase of local and non-local energetic disorder that is determined by structural disorder. This energetic disorder is weakly sensitive to the distance between dopant ions and polymer chains, R_{dop}.

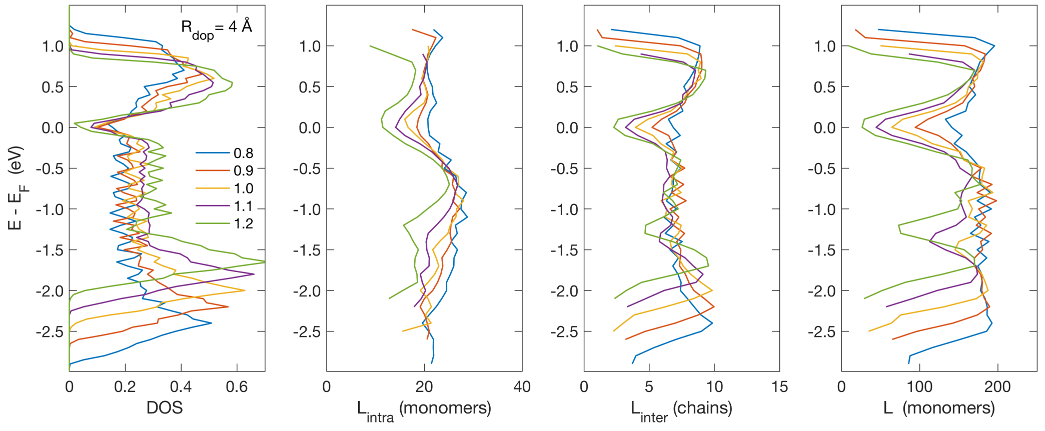

Figure S36: Electronic properties as a function of the Huang-Rhys factor S (see legend), determining the renormalization of charge transfer integrals due to high-frequency vibrations. Results are shown as in Figure 29. The hoppings renormalization factor is a crucial parameter controlling the interplay between competing interactions, namely charge delocalization and Coulomb interactions, with large S values leading to more localized states. Results obtained for $g_{\pi-\pi}$=10 % and R_{dop}=4 Å.

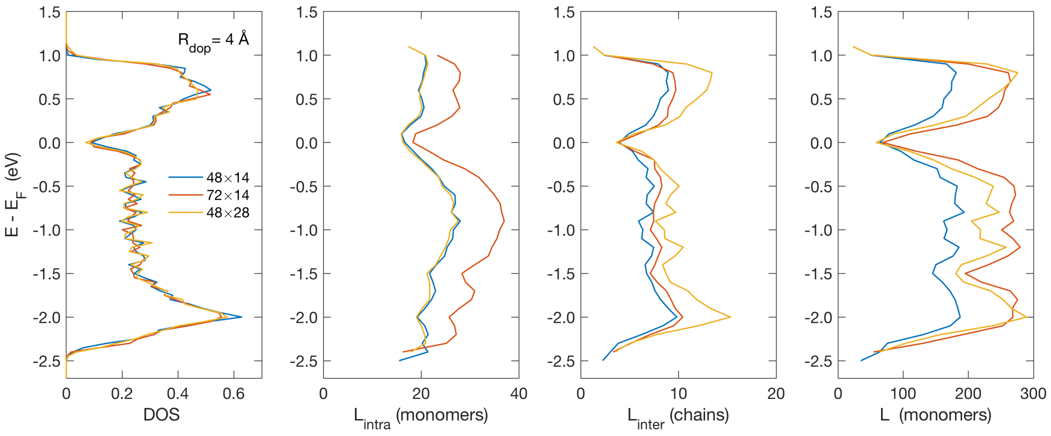

Figure S37: Electronic properties as a function of the system size (see legend). Results are shown as in Figure 29. The DOS and the delocalization of states at E_F are reasonably converged with respect to the size of the lattice, even for the present system, characterized by low energetic disorder. Results obtained for $g_{\pi-\pi}$=10 % and R_{dop}=4 Å.

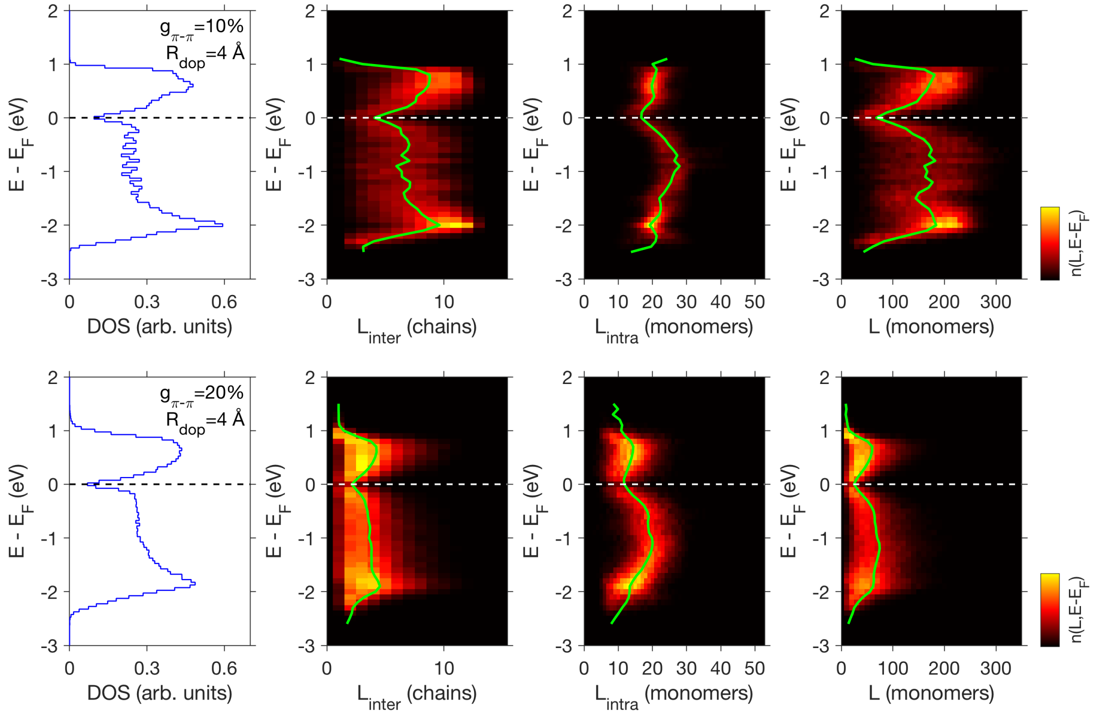

Figure S38: Electronic properties for two systems characterized by $g_{\pi-\pi}$=10 % (top panels) and $g_{\pi-\pi}$=20 % (bottom panels). Panels from left to right show the DOS and charge delocalization observables (L_{intra}, L_{inter}, L). The latter are presented as two-dimensional histograms, where the distribution of eigenstates is resolved in energy and localization length. Green lines show the localization length, averaged over energy intervals. These data show that the spread of the wavefunctions largely fluctuates over the whole energy range, except around E_F, where the states are more localized.

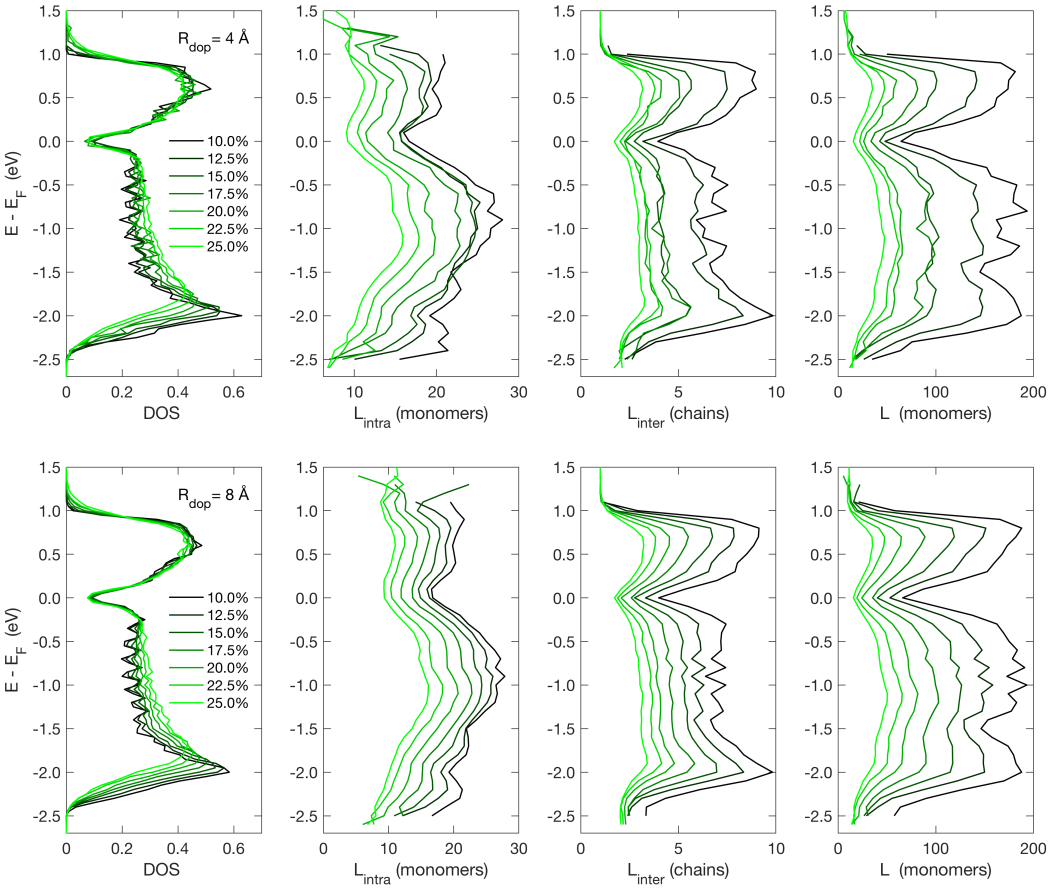

Figure S 39: Electronic properties as a function of the key paracrystallinity parameter $g_{\pi-\pi}$ (see legend), for $R_{\rm dop}$=4 Å(top panels) and $R_{\rm dop}$=8 Å(bottom panels). Results are shown as in Figure 29. This full data set shows how paracrystallinity is the crucial parameter that determines the spread on the wavefunctions in the whole energy range, and at E_F in particular. On the other hand, the DOS is marginally affected by paracrystallinity.

References

[1] Ian E. Jacobs, Yue Lin, Yuxuan Huang, Xinglong Ren, Dimitrios Simatos, Chen Chen, Dion Tjhe, Martin Statz, Lianglun Lai, Peter A. Finn, William G. Neal, Gabriele D'Avino, Vincent Lemaur, Simone Fratini, David Beljonne, Joseph Strzalka, Christian B. Nielsen, Stephen Barlow, Seth R. Marder, Iain McCulloch, and Henning Sirringhaus. High efficiency ion exchange doping of conducting polymers. *Advanced Materials*, 2021.

[2] Keehoon Kang, Shun Watanabe, Katharina Broch, Alessandro Sepe, Adam Brown, Iyad Nasrallah, Mark Nikolka, Zhuping Fei, Martin Heeney, and Daisuke Matsumoto. 2d coherent charge transport in highly ordered conducting polymers doped by solid state diffusion. *Nature materials*, 15(8):896–902, 2016.

[3] Amer Hamidi-Sakr, Laure Biniek, Jean-Louis Bantignies, David Maurin, Laurent Herrmann, Nicolas Leclerc, Patrick Lévêque, Vishnu Vijayakumar, Nicolas Zimmermann, and Martin Brinkmann. A versatile method to fabricate highly in-plane aligned conducting polymer films with anisotropic charge transport and thermoelectric properties: The key role of alkyl side chain layers on the doping mechanism. *Advanced Functional Materials*, 27(25):1700173–n/a, 2017.

[4] D Tyler Scholes, Patrick Y Yee, Jeffrey R Lindemuth, Hyeyeon Kang, Jonathan Onorato, Raja Ghosh, Christine K Luscombe, Frank C Spano, Sarah H Tolbert, and Benjamin J Schwartz. The effects of crystallinity on charge transport and the structure of sequentially processed f4tcnq-doped conjugated polymer films. *Advanced Functional Materials*, 2017.

[5] R. Joseph Kline, Dean M. DeLongchamp, Daniel A. Fischer, Eric K. Lin, Lee J. Richter, Michael L. Chabinyc, Michael F. Toney, Martin Heeney, and Iain McCulloch. Critical role of side-chain attachment density on the order and device performance of polythiophenes. *Macromolecules*, 40(22):7960–7965, 10 2007.

[6] A. C. Mayer, Michael F. Toney, Shawn R. Scully, Jonathan Rivnay, Christoph J. Brabec, Marcus Scharber, Marcus Koppe, Martin Heeney, Iain McCulloch, and Michael D. McGehee. Bimolecular crystals of fullerenes in conjugated polymers and the implications of molecular mixing for solar cells. *Advanced Functional Materials*, 19(8):1173–1179, 2020/10/03 2009.

[7] Ian E. Jacobs, Camila Cendra, Thomas F. Harrelson, Zaira I. Bedolla Valdez, Roland Faller, Alberto Salleo, and Adam J. Moulé. Polymorphism controls the degree of charge transfer in a molecularly doped semiconducting polymer. *Materials Horizons*, 5(4):655–660, 2018.

[8] Yu Yamashita, Junto Tsurumi, Masahiro Ohno, Ryo Fujimoto, Shohei Kumagai, Tadanori Kurosawa, Toshihiro Okamoto, Jun Takeya, and Shun Watanabe. Efficient molecular doping of polymeric semiconductors driven by anion exchange. *Nature*, 572(7771):634–638, 2019.

[9] Colette Moreau and Gérard Douhéret. Thermodynamic and physical behaviour of water + acetonitrile mixtures. dielectric properties. *The Journal of Chemical Thermodynamics*, 8(5):403–410, 1976.

[10] Elayne M. Thomas, Kelly A. Peterson, Alex H. Balzer, Dakota Rawlings, Natalie Stingelin, Rachel A. Segalman, and Michael L. Chabinyc. Effects of counter-ion size on delocalization of carriers and stability of doped semiconducting polymers. *Advanced Electronic Materials*, n/a(n/a):2000595, 2020/11/03 2020.

[11] Shu-Jen Wang, Deepak Venkateshvaran, M. R. Mahani, Uday Chopra, Erik R. McNellis, Riccardo Di Pietro, Sam Schott, Angela Wittmann, Guillaume Schweicher, Murat Cubukcu, Keehoon Kang, Remington Carey, Thomas J. Wagner, Janis N. M. Siebrecht, Daniel P. G. H. Wong, Ian E. Jacobs, Razan O. Aboljadayel, Adrian Ionescu, Sergei A. Egorov, Sebastian Mueller, Olga Zadvorna, Piotr Skalski, Cameron Jellett, Mark Little, Adam Marks, Iain McCulloch, Joerg Wunderlich, Jairo Sinova, and Henning Sirringhaus. Long spin diffusion lengths in doped conjugated polymers due to enhanced exchange coupling. *Nature Electronics*, 2(3):98–107, 2019.

[12] Stefanie Winkler, Patrick Amsalem, Johannes Frisch, Martin Oehzelt, Georg Heimel, and Norbert Koch. Probing the energy levels in hole-doped molecular semiconductors. *Materials Horizons*, 2(4):427–433, 2015.

[13] Annabel R. Chew, Raja Ghosh, Zhengrong Shang, Frank C. Spano, and Alberto Salleo. Sequential doping reveals the importance of amorphous chain rigidity in charge transport of semi-crystalline polymers. *The Journal of Physical Chemistry Letters*, 8(20):4974–4980, 10 2017.

[14] Ian E. Jacobs, Faustine Wang, Nema Hafezi, Cristina Medina-Plaza, Thomas F. Harrelson, Jun Li, Matthew P. Augustine, Mark Mascal, and Adam J. Moulé. Quantitative dedoping of conductive polymers. *Chemistry of Materials*, 29(2):832–841, 01 2017.

[15] Justin E. Cochran, Matthias J. N. Junk, A. M. Glaudell, P. Levi Miller, John S. Cowart, Michael F. Toney, Craig J. Hawker, Bradley F. Chmelka, and Michael L. Chabinyc. Molecular interactions and ordering in electrically doped polymers: Blends of pbttt and f(4)tcnq. *Macromolecules*, 47(19):6836–6846, 2014.

[16] Renxuan Xie, Albree R. Weisen, Youngmin Lee, Melissa A. Aplan, Abigail M. Fenton, Ashley E. Masucci, Fabian Kempe, Michael Sommer, Christian W. Pester, Ralph H. Colby, and Enrique D. Gomez. Glass transition temperature from the chemical structure of conjugated polymers. *Nature Communications*, 11(1):893, 2020.

[17] Neal A. Yakelis and Robert G. Bergman. Safe preparation and purification of sodium tetrakis[(3,5-trifluoromethyl)phenyl]borate (nabarf24): Reliable and sensitive analysis of water in solutions of fluorinated tetraarylborates. *Organometallics*, 24(14):3579–3581, 07 2005.

[18] Jing Li, Gabriele D'Avino, Anton Pershin, Denis Jacquemin, Ivan Duchemin, David Beljonne, and Xavier Blase. Correlated electron-hole mechanism for molecular doping in organic semiconductors. *Phys. Rev. Materials*, 1:025602, Jul 2017.

[19] Simone Fratini, Didier Mayou, and Sergio Ciuchi. The transient localization scenario for charge transport in crystalline organic materials. *Advanced Functional Materials*, 26(14):2292–2315, 2016.

[20] S. Fratini and S. Ciuchi. Dynamical localization corrections to band transport. *Phys. Rev. Research*, 2:013001, Jan 2020.

[21] C. Reinhold, E. W. Fischer, and A. Peterlin. Evaluation of small-angle x-ray scattering of polymers. *Journal of Applied Physics*, 35(1):71–74, 1964.

[22] I. H. Hall, E. A. Mahmoud, P. D. Carr, and Y. D. Geng. Small-angle-x-ray scattering by crystalline polymer fibres 1. experimental method and investigation of the linear paracrystalline model. *Colloid and Polymer Science*, 265:383–393, 1987.

[23] Anas Abutaha, Pawan Kumar, Erol Yildirim, Wen Shi, Shuo-Wang Yang, Gang Wu, and Kedar Hippalgaonkar. Correlating charge and thermoelectric transport to paracrystallinity in conducting polymers. *Nature Communications*, 11(1):1737, 2020.

[24] Frank Neese. The orca program system. *WIREs Comput. Mol. Sci.*, 2(1):73–78, 2012.

[25] Deepak Venkateshvaran, Mark Nikolka, Aditya Sadhanala, Vincent Lemaur, Mateusz Zelazny, Michal Kepa, Michael Hurhangee, Auke Jisk Kronemeijer, Vincenzo Pecunia, and Iyad Nasrallah. Approaching disorder-free transport in high-mobility conjugated polymers. *Nature*, 515(7527):384–388, 2014.

[26] Vincent Lemaur, Jérôme Cornil, Roberto Lazzaroni, Henning Sirringhaus, David Beljonne, and Yoann Olivier. Resilience to conformational fluctuations controls energetic disorder in conjugated polymer materials: Insights from atomistic simulations. *Chemistry of Materials*, 31(17):6889–6899, 2019.

[27] Stewart J. Clark, Matthew D. Segall, Chris J. Pickard, Phil J. Hasnip, Matt I. J. Probert, Keith Refson, and Mike C. Payne. First principles methods using castep. *Zeitschrift für Kristallographie - Crystalline Materials*, 220(5-6):567 – 570, 01 May. 2005.

[28] K. Hannewald, V. M. Stojanović, J. M. T. Schellekens, P. A. Bobbert, G. Kresse, and J. Hafner. Theory of polaron bandwidth narrowing in organic molecular crystals. *Phys. Rev. B*, 69:075211, 2004.

[29] Demetrio A da Silva Filho, Veaceslav Coropceanu, Denis Fichou, Nadine E Gruhn, Tonja G Bill, Johannes Gierschner, Jérôme Cornil, and Jean-Luc Brédas. Hole-vibronic coupling in oligothiophenes: impact of backbone torsional flexibility on relaxation energies. *Philosophical Transactions of the Royal Society A: Mathematical, Physical and Engineering Sciences*, 365(1855):1435–1452, 2007.
